# Supplementary material for: Parents and carers’ experiences of seeking health information and support online for long-term physical childhood conditions: a systematic review and thematic synthesis of qualitative research
Source: BMJ Open. 2020 Dec 28;10(12):e042139. doi: 10.1136/bmjopen-2020-042139 (PMC7772330; doi:10.1136/bmjopen-2020-042139)
Supplement: Author's manuscript [file bmjopen-2020-042139.draft_revisions.pdf]

BMJ Open is committed to open peer review. As part of this commitment we make the peer review history of every article we publish publicly available.

When an article is published we post the peer reviewers' comments and the authors' responses online. We also post the versions of the paper that were used during peer review. These are the versions that the peer review comments apply to.

The versions of the paper that follow are the versions that were submitted during the peer review process. They are not the versions of record or the final published versions. They should not be cited or distributed as the published version of this manuscript.

BMJ Open is an open access journal and the full, final, typeset and author-corrected version of record of the manuscript is available on our site with no access controls, subscription charges or pay-per-view fees (http://bmjopen.bmj.com).

If you have any questions on BMJ Open's open peer review process please email info.bmjopen@bmj.com

BMJ Open

Parents and carers' experiences of seeking health information and support online for long-term physical childhood conditions: A systematic review and thematic synthesis of qualitative research

Journal:	*BMJ Open*
Manuscript ID	bmjopen-2020-042139
Article Type:	Original research
Date Submitted by the Author:	30-Jun-2020
Complete List of Authors:	Treadgold, Bethan; University of Southampton Faculty of Medicine, Primary Care, Population Sciences and Medical Education<br>Teasdale, Emma; University of Southampton Faculty of Medicine, Primary Care, Population Sciences and Medical Education<br>Muller, Ingrid; University of Southampton Faculty of Medicine, Primary Care, Population Sciences and Medical Education<br>Roberts, Amanda; University of Nottingham Faculty of Medicine and Health Sciences, Centre of Evidence-Based Dermatology<br>Coulson, Neil; University of Nottingham Faculty of Medicine and Health Sciences, Division of Rehabilitation, Ageing and Wellbeing<br>Santer, Miriam; University of Southampton Faculty of Medicine, Primary Care, Population Sciences and Medical Education
Keywords:	PAEDIATRICS, QUALITATIVE RESEARCH, World Wide Web technology < BIOTECHNOLOGY & BIOINFORMATICS
Note: The following files were submitted by the author for peer review, but cannot be converted to PDF. You must view these files (e.g. movies) online.	
Figure 1_PRISMA flow diagram of systematic search and study selection.xps	

SCHOLARONE™
Manuscripts

I, the Submitting Author has the right to grant and does grant on behalf of all authors of the Work (as defined in the below author licence), an exclusive licence and/or a non-exclusive licence for contributions from authors who are: i) UK Crown employees; ii) where BMJ has agreed a CC-BY licence shall apply, and/or iii) in accordance with the terms applicable for US Federal Government officers or employees acting as part of their official duties; on a worldwide, perpetual, irrevocable, royalty-free basis to BMJ Publishing Group Ltd ("BMJ") its licensees and where the relevant Journal is co-owned by BMJ to the co-owners of the Journal, to publish the Work in this journal and any other BMJ products and to exploit all rights, as set out in our licence.

The Submitting Author accepts and understands that any supply made under these terms is made by BMJ to the Submitting Author unless you are acting as an employee on behalf of your employer or a postgraduate student of an affiliated institution which is paying any applicable article publishing charge ("APC") for Open Access articles. Where the Submitting Author wishes to make the Work available on an Open Access basis (and intends to pay the relevant APC), the terms of reuse of such Open Access shall be governed by a Creative Commons licence – details of these licences and which Creative Commons licence will apply to this Work are set out in our licence referred to above.

Other than as permitted in any relevant BMJ Author's Self Archiving Policies, I confirm this Work has not been accepted for publication elsewhere, is not being considered for publication elsewhere and does not duplicate material already published. I confirm all authors consent to publication of this Work and authorise the granting of this licence.

For peer review only

Parents and carers' experiences of seeking health information and support online for long-term physical childhood conditions:

A systematic review and thematic synthesis of qualitative research

Bethan M. Treadgold[1], Emma J. Teasdale[1], Ingrid Muller[1], Amanda Roberts[2,] Neil S. Coulson[3], Miriam Santer[1]

[1] School of Primary Care, Population Health and Medical Education, University of Southampton, UK

[2] Nottingham Support Group for Carers of Children with Eczema, Nottingham, UK

[3] School of Medicine, Division of Rehabilitation, Ageing and Wellbeing, University of Nottingham, UK

For peer review only

Word count: 3533 (Including everything from Introduction to Conclusion except for tables and figures. In-text quotations and table/figure labels are included.)

Corresponding author:

Bethan M. Treadgold

School of Primary Care, Population Health and Medical Education, University of Southampton, UK

b.m.treadgold@southampton.ac.uk

For peer review only - http://bmjopen.bmj.com/site/about/guidelines.xhtml

Abstract

Objective To systematically review and synthesise qualitative research exploring parents/carers' experiences of seeking online information and support for long-term physical childhood conditions.

Design Systematic review and thematic synthesis of qualitative research.

Data sources Medline, CINAHL, Embase, PsycINFO, and The International Bibliography of the Social Sciences were searched from inception to September 2019. We used thematic synthesis to analyse the findings.

Eligibility criteria Primary research papers presenting qualitative data collection and analysis, focusing on parents/carers' experiences of seeking health information and support from online resources for long-term physical childhood health conditions. No language restrictions were placed.

Results 23 studies from seven countries met inclusion criteria and were included in the synthesis. Included studies presented data collected through interviews/focus groups with 559 parents/carers; open-ended/essay surveys with 26 parents/carers; and 2407 messages from online support groups. Parents/carers developed a variety of strategies to obtain information and support online, based on personal preferences, appraisal of trustworthiness, perceived needs, and previous experiences online. In most cases, they sought the benefits of online information and support, which included feeling empowered with further medical knowledge and perceived control, reassurance and validation from online communities. Some concerns and perceived risks were discussed, which often stemmed from prior unsatisfactory experiences of seeking information and support online, consultations with health professionals, and seeing distressing stories online.

Conclusion Most parents/carers were successful in obtaining information and support online. Many continued to share experiences with other parents/carers online. The need for information was particularly apparent early after diagnosis of the condition, whereas the need for peer-support continued. The potential concerns and perceived risks with information and support online were especially apparent amongst parents/carers of children with life-limiting long-term conditions. Findings may be useful for health professionals to facilitate discussions regarding use of online resources, and researchers designing online health resources for parents/carers.

PROSPERO registration number The protocol for this study was registered with PROSPERO in May 2018 [CRD42018096009].

For peer review only - http://bmjopen.bmj.com/site/about/guidelines.xhtml

Strengths and limitations of this study

- Comprehensive study summarising and providing new insights into the online experiences of parents/carers of children with long-term physical conditions
- Convenient for health professionals and researchers interacting with this population
- Excludes the online experiences of parents/carers of children with acute, mental health, or developmental conditions, and otherwise healthy children.

For peer review only

For peer review only - http://bmjopen.bmj.com/site/about/guidelines.xhtml

Background

Caring for a child with a long-term condition can be challenging for parents and carers, requiring ongoing management with regular treatment use, and often complicated, time-consuming and emotionally-laden everyday routines (1). Seeking to maintain treatment and care regimes, dealing with social and financial constraints, whilst balancing the needs of the whole family increase these challenges (2). For the remainder of the paper, parents and carers are collectively referred to as 'parents'.

As a source of health information, the Internet is convenient, instant and can help enhance knowledge and understanding of a condition (3). As a source of support, the Internet also enables parents to exchange stories with other parents with similar experiences. This commonly occurs through asynchronous discussion platforms (4), although synchronous (real-time) discussions through online platforms are also increasingly popular. Reviews of qualitative research have explored how the Internet influences peoples' health experiences (5, 6). Experiences include obtaining health information to understand illness, redressing offline information and knowledge deficits, discussing experiences with health services, building connections with others, feeling supported and validated, releasing emotions and offline frustrations, and learning self-management behaviours from others. Other reviews of qualitative research surrounding this area have explored the views of physicians regarding social media interventions to support child health (7), and experiences with health information online for carers of adults living with cancer (8). However, no systematic review has been conducted that synthesises the qualitative research on online experiences of parents of children with long-term physical conditions.

Aim

This review seeks to explore parents' experiences of seeking information and support online for long-term physical childhood conditions.

Method

This study follows The Centre for Reviews and Disseminations guidance for undertaking reviews in health care (9), and the ENTREQ guidelines for reporting the synthesis of qualitative research (10). The protocol for this study was registered with PROSPERO in May 2018 [CRD 2016018096009].

Eligibility criteria

We sought studies that explored the experiences of parents of children with any long-term physical condition(s) around using the Internet for information and support (Table 1). To be eligible for inclusion, studies had to present qualitative data collection and analysis methods, and qualitative data. Studies that used a mixed methodology were included if they comprised a qualitative component substantive enough to perform a qualitative analysis.

	Inclusion criteria	Exclusion criteria

For peer review only - http://bmjopen.bmj.com/site/about/guidelines.xhtml

**Setting**	• Online health information and support resources (e.g. websites, forums, social media pages, blogs)	• Primarily offline health information and support resources (e.g. family, friends, books, leaflets)
**Design**	• Qualitative data collection methods (or mixed-methods studies involving qualitative data) (e.g. interviews), qualitative data analysis methods (e.g. thematic analysis) and presented qualitative data (e.g. themes, narratives and quotations)<br>• Primary research (e.g. full research papers, feasibility studies and pilot studies)	• Did not present the qualitative study criteria<br>• Not primary research (e.g. reviews, protocols and commentaries)
**Population**	• Parents and carers of children living with long-term physical health conditions	• Not parents or carers (e.g. family members without caring responsibility for the child, health professionals and teachers)
**Condition**	• Long-term physical health conditions<br>- Definition: A long-term or chronic physical health condition is a condition that requires ongoing management which cannot currently be cured but can be controlled with regular adherence to treatment (11)	• Acute conditions (e.g. common cold)<br>• Mental health conditions (e.g. depression)<br>• Developmental disorders of learning, language and cognition (e.g. Attention Deficit Hyperactivity Disorder)<br>• Healthy populations or health-promoting related studies (e.g. diet, physical activity, obesity)
**Outcome**	• Parents and primary carers' experiences	• Not parents' experiences (e.g. patterns of Internet usage derived from quantitative studies)

Table 1. Eligibility criteria.

Search strategy

Five electronic databases were searched (Medline, CINAHL, Embase, PsycINFO, and The International Bibliography of the Social Sciences) from the earliest date possible in each database to September 2019. The Cochrane Library of Systematic Reviews and PROSPERO were also searched to check for any ongoing similar reviews. A comprehensive search strategy was developed for each database (Table 2). Search terms seeking to identify only qualitative research were initially included in search strategies, but we found they did not improve the search results so were subsequently removed from search strategies. Others have similarly found subject-specific search strategies rather than methodology-based strategies to be more effective in identifying all relevant studies reporting qualitative findings (12).

For peer review only - http://bmjopen.bmj.com/site/about/guidelines.xhtml

Database	Internet search terms	Parent/carer search terms	Use of online health information search terms	Paediatric search terms
**MEDLINE**	1. exp Internet/<br>2. forum*.mp.<br>3. facebook.mp.<br>4. social media.mp.<br>5. blog*.mp.<br>6. Internet.mp.<br>7. (web or online).mp.	1. parents/ or fathers/ or mothers/ or single parent/<br>2. Caregivers/<br>3. Legal Guardians/<br>4. (parent or parents).mp.<br>5. caregiver*.mp.<br>6. carer*.mp.<br>7. guardian*.mp.	1. Help-seeking Behaviour/<br>2. health information.mp.<br>3. Self-Help Groups/<br>4. self help.mp.<br>5. exp Health Education/<br>6. patient information.mp.<br>7. medical information.mp.<br>8. health information.mp.	1. Child Health/<br>2. Child/<br>3. Pediatrics/<br>4. (child or children or paediatric* or pediatric*).mp.
**CINAHL**	1. SU Internet<br>2. forum<br>3. facebook<br>4. social media<br>5. blog*<br>6. Internet	1. SU (parent* OR father* OR mother* OR single parent*)<br>2. SU caregivers<br>3. SU legal guardians<br>4. (parent OR parents)<br>5. caregiver*<br>6. carer*<br>7. guardian*	1. SU help seeking behaviour<br>2. health information<br>3. SU self help groups<br>4. self help<br>5. SU health education<br>6. patient information<br>7. medical information<br>8. health information	1. SU child health<br>2. SU child<br>3. SU paediatrics<br>4. (child OR children OR paediatric* OR pediatric*)
**Embase**	1. exp Internet/<br>2. forum*.mp.<br>3. facebook.mp.<br>4. social media.mp.<br>5. blog*.mp.<br>6. Internet.mp.<br>7. (web or online).mp	1. parents/ or fathers/ or mothers/ or single parent/<br>2. Caregivers/<br>3. Legal Guardians/<br>4. (parent or parents).mp.<br>5. caregiver*.mp.<br>6. carer*.mp.<br>7. guardian*.mp	1. Help-seeking Behaviour/<br>2. health information.mp.<br>3. Self-Help Groups/<br>4. self help.mp.<br>5. exp Health Education/<br>6. patient information.mp.<br>7. medical information.mp.<br>8. health information.mp.	1. Child Health/<br>2. Child/<br>3. Pediatrics/<br>4. (child or children or paediatric* or pediatric*).mp.
**PsycINFO**	1. SU Internet<br>2. forum<br>3. facebook<br>4. social media<br>5. blog*<br>6. Internet	1. SU (parent* OR father* OR mother* OR single parent*)<br>2. SU caregivers<br>3. SU legal guardians<br>4. (parent OR parents)<br>5. caregiver*<br>6. carer*<br>7. guardian*	1. SU help seeking behaviour<br>2. health information<br>3. SU self help groups<br>4. self help<br>5. SU health education<br>6. patient information<br>7. medical information<br>8. health information	1. SU child health<br>2. SU child<br>3. SU paediatrics<br>4. (child OR children OR paediatric* OR pediatric*)

For peer review only

For peer review only - http://bmjopen.bmj.com/site/about/guidelines.xhtml

**International Bibliography of Social Science**	1. Ab (Internet* OR forum* OR facebook OR social media OR blog* OR web* OR online)	1. Ab (parent* OR father* OR mother* OR caregiver* OR guardian* OR carer*)	1. Ab (help seeking behaviour* OR health information OR self help group* OR self help* OR health education OR patient information OR medical information)	1. Ab (child health OR child* OR paediatric* OR pediatric*)

Table 2. Search terms and syntax for each database.

For peer review only

For peer review only - http://bmjopen.bmj.com/site/about/guidelines.xhtml

Study screening methods

One author (BT) screened the titles and abstracts of all identified papers against the inclusion criteria. Duplicates of papers, and those that failed to meet the inclusion criteria were eliminated during this stage. A second author (ET) screened 20% of the titles and abstracts to check for consistency. Full papers for the remaining identified studies were retrieved and assessed for eligibility (see figure 1).

Quality appraisal and data extraction

One author (BT) extracted information from the included studies and tabulated it. Assessors (BT, IM, MS, ET) independently assessed the quality of the included papers, using the Critical Appraisal Skills Programme (CASP) quality assessment tool for qualitative research (13). Any discrepancies were discussed with the research team.

Thematic Synthesis

We followed thematic synthesis for the analysis of the included studies (14), as this method has been successfully used for synthesising qualitative research in systematic reviews in health (15-20). Thematic synthesis consists of three stages. In stages 1 and 2 we explored and described the data. One author (BT) read and re-read included papers, then inductively coded each line of the studies' findings according to their content and meaning to develop an initial coding frame. Participants' original data excerpts, as well as the authors' descriptions of findings, were coded in the latest version of NVivo. The coding frame was then discussed and refined within the research team (BT, ET, IM, AR, NC, MS). In stage 3 we aimed to 'go beyond' individual study findings through developing descriptive themes initially and, subsequently, generating analytical themes.

Patient and Public Involvement

We involved an experienced public contributor with experience of eczema (AR) in the analysis and write-up stages of the review.

Findings

Study selection

The database searches resulted in 2982 records after excluding duplicates. A final 23 (21-43) studies met all inclusion criteria and were included for thematic synthesis (Figure 1).

Study characteristics

All studies were published in English between 2004 and 2019, with the majority of studies (n = 14) published since 2016. 11 studies were conducted in the Americas (USA (6), Canada (4), Brazil (1)), 10 studies were conducted in Europe (UK (5), Sweden (2), The Netherlands (2), and Norway (1)), and 2 studies were conducted in Australia. The 23 studies presented the experiences of 559 parents captured through interviews and focus groups, 26 parents through open-ended surveys or essay surveys, and analyses of 2407 messages that had been

For peer review only - http://bmjopen.bmj.com/site/about/guidelines.xhtml

posted by parents in online support groups, though it should be noted that not all studies provided sample size information (Table 3).

For peer review only

For peer review only - http://bmjopen.bmj.com/site/about/guidelines.xhtml

Authors of study (Country) (ref.)	Aim(s)	Method of data collection (sample size)	Method of data analysis (as written by the authors)	Condition(s)
**Alsem et al., 2019 (The Netherlands) (21)**	To explore parental and physicians' experiences with the WWW-roadmap, as well as differences between parents who had used the WWW-roadmap to prepare for a consultation with their rehabilitation physician and parents who had not, in terms of empowerment and self-efficacy, parental and professional satisfaction with the consultation and family centredness of care.	Semi-structured interviews (sample size not stated)	Theoretical thematic analysis	Disabilities: Cerebral palsy , Neuromuscular, Metabolic disorder, Genetic disorder, Spina bifida, Various syndromes (e.g., Down's), Movement disorder, Unknown, Other
**Benedicta et al., 2019 (Australia) (22)**	To understand how parents search for and appraise the relevance and credibility of online health information regarding their child, and how they use this information in order to help them make better health decisions for their child.	Think aloud task of searching online health info and semi-structured interviews (22)	Observation checklist for the think aloud task; inductive thematic analysis for the interviews	'Subacute' conditions
**Canty et al., 2019 (Canada) (23)**	To qualitatively and quantitatively assess the use of social media as an information-sharing and support-seeking tool by patients and caregivers**.**	Analysis of social media postings (sample size not stated)	Modified thematic analysis	Cerebral palsy
**Gage-Bouchard et al., 2019 (USA) (24)**	To examine the reasons paediatric cancer caregivers engage with cancer-related information on SNS and how caregivers appraise and assess the credibility of cancer-related information they encounter on this platform.	Semi-structured interviews (40)	Thematic analysis	Cancer
**Aston et al., 2018 (UK) (25)**	To explore the treatment-related experiences when children and young people take regular prescribed medication.	Semi-structured interviews (23 parents)	Thematic analysis	In-patients taking two or more prescribed medications concurrently at home, prior to admission, for 6 weeks or longer.<br>Names of conditions not provided.

For peer review only - http://bmjopen.bmj.com/site/about/guidelines.xhtml

**Hinton et al., 2018 (UK) (26)**	To explore parents' experiences and perspectives of having a baby who needs early abdominal surgery; identify the questions and problems that matter to parents during and after their pregnancy and infant's surgery and identify the long-term impact on parents and families.	Interviews (starting unstructured then semi-structured) (44)	Interpretative thematic analysis using a modified grounded theory approach, with constant comparison and exploration of deviant cases	Infants who needed abdominal surgery in the first year of life
**Holtz et al., 2018 (USA) (27)**	To develop a website resource, as an initial step in a larger intervention, for parents with a child with Type 1 Diabetes.	Focus groups (3 parents), interviews (3)	Iterative thematic analysis	Type 1 Diabetes
**Perez et al., 2018 (USA) (28)**	Stated as RQs: RQ1: What types of uncertainty do parents of a child with Type 1 Diabetes report experiencing?; RQ2: How do parents manage this uncertainty?	In-depth interviews (29)	Thematic analysis	Type 1 Diabetes
**Wright 2018 (USA) (29)**	To qualitatively explore the informal learning experiences of members of an online social media group hosted by Facebook.	Semi-structured interviews (25) and analyses of 604 postings (original and comments) created by members of the online social media	Several Grounded Theory methodologies	Gastro-oesophageal reflux disease
**Rehman et al., 2018 (Canada) (30)**	To establish the dominant themes of tweets posted by parents of children diagnosed with cancer and to situate the themes within a theoretical framework that describes the phenomenon of information sharing within a non-work context.	Analysis of the content of 1700 tweets from the Twitter accounts of 15 recruited participants	A deductive subjective interpretation	Cancer
**Tracey et al., 2018 (Australia) (31)**	To explore how parents acquire information to enhance their understanding of their child's disability and individualised funding schemes.	Focus groups (56 parents, 13 groups)	A a two stage, mixed method sequential approach which followed an initial survey	'disability'
**Alsem et al., 2017 (The Netherlands) (32)**	To explore the experiences of Dutch parents of children with disabilities as regards their information needs, and to describe the process of seeking and evaluating information and the different sources of information.	Semi-structured interviews (15)	Open coding thematic analysis	Cerebral palsy, GMFCSa 2–5, rare disorders (e.g. genetic syndromes and metabolic and mitochondrial diseases)
**Kirk & Milnes, 2016 (UK) (33)**	To explore how online peer support is used by young people and parents to support self-care in relation to cystic fibrosis.	Analysis of discussion group postings (103)	Inductive grounded theory	Cystic fibrosis

For peer review only - http://bmjopen.bmj.com/site/about/guidelines.xhtml

**Vogel et al., 2016 (Canada) (34)**	To explore parental usage of the National Kidney Foundation website, track visitor behaviour, evaluate usability and design, establish ways to improve user experience, and identify ways to redesign the website.	Mixed methods: Google analytic usage reports and focus groups (4)	Inductive content analysis and constant comparative approach	A condition requiring neurosurgery
**Santer et al., 2015 (UK) (35)**	To explore parents and carers' experiences of searching for information about childhood eczema on the Internet.	Semi-structured interviews (31)	Content analysis and constant comparative approach	Eczema
**Cargnin Plimentel et al., 2013 (Brazil) (36)**	To describe the experience of patients with cystic fibrosis and their families using the Internet as a means for information, interaction and exchange of experiences with this disease.	Questionnaire (7)	Content analysis	Cystic fibrosis
**Nordfeldt et al., 2013 (Sweden) (37)**	To explore parents' views on their information-seeking, Internet use, and social networking online, and to identify implications for the future development of Internet use in this respect.	Focus groups (27)	Inductive qualitative content analysis	Type 1 Diabetes
**Gage & Panagakis, 2012 (USA) (38)**	To examine how parents think about, evaluate, access and use the Internet to seek information related to their child's cancer.	Mixed methods: Survey and structured interviews (41)	Grounded theory	Cancer
**Gundersen, 2011 (Norway) (39)**	To explore how the Internet can function as a resource that parents can draw on to cope with their situation.	Semi-structured interviews (10)	Not a specified method but looks like thematic analysis with comparative approach based on theorist	Rare genetic disorders that entail developmental and/or physical problems (e.g. allergies and epilepsy, and two had severe physical and learning problems)
**Stewart et al., 2011 (Canada) (40)**	To gather parents' preferences of support to design an innovative online peer support and test it.	Semi-structured interviews (44)	Deductive thematic content analysis	Asthma and allergies
**Nordfeldt et al., 2010 (Sweden) (41)**	To explore patients' and parents' attitudes toward a local Web 2.0 portal tailored to young patients with type 1 diabetes and their parents.	Essays (19)	Content analysis	Type 1 Diabetes

For peer review only - http://bmjopen.bmj.com/site/about/guidelines.xhtml

**Roche & Skinner, 2009 (USA) (42)**	To describe how parents of a child referred for genetic services searched the Internet for information, summarise how they interpreted and evaluated the information they obtained, and identify barriers that they encountered.	Semi-structured interviews (100)	Constant comparative method	Genetic
**Nettleton et al., 2004 (UK) (43)**	To explore parents and children's experiences of using the Internet for chronic childhood conditions.	Qualitative interviews (69)	Strategic comparisons approach'	Eczema, asthma, diabetes

Table 3. Summaries of the final 23 papers for thematic synthesis.

For peer review only

For peer review only - http://bmjopen.bmj.com/site/about/guidelines.xhtml

Quality of studies

The quality assessment of the included studies revealed that all studies met most quality indicators. All studies had clear statements of the aims of the research, qualitative methodology was appropriate in all studies and was collected in a way that addressed the aims. We did not exclude any studies based on quality of reporting.

Synthesis of results

The papers included in this review broadly focused on how parents of children with long-term physical conditions use online resources for health information and support, and what they gain (or not) from using online resources. By synthesising the papers' findings, we interpreted three main analytical themes: 1) Developing personal strategies for obtaining and appraising the online world; 2) Feeling empowered and supported by the online world; 3) Perceived risk and concerns about the online world.

Figure 2 illustrates the analytical themes and codes, and table 4 lists all analytical and descriptive themes, along with references to the relevant papers.

For peer review only

For peer review only - http://bmjopen.bmj.com/site/about/guidelines.xhtml

Analytical theme	Descriptive theme	Paper references [total number]
**Developing personal strategies for obtaining and appraising the online world**		
	**Personal preferences**	22, 24, 27, 28, 29, 32, 33, 34, 35, 36, 37, 38, 39, 40, 41, 42, 43 **[17]**
	**Searching methods**	22, 24, 29, 31, 32, 34, 35, 37, 38, 39, 42, 43 **[12]**
	**Appraisal of trustworthiness and credibility**	22, 24, 27, 28, 29, 31, 32, 34, 35, 36, 37, 38, 43 **[13]**
	**Perceived need of information and support**	21, 22, 23, 26, 27, 28, 29, 33, 34, 35, 36, 37, 38, 39, 40, 41, 42, 43 **[18]**
	**Past positive experiences online**	21, 22, 24, 27, 28, 29, 31, 35, 36, 37, 38, 39, 40, 41, 42, 43 **[16]**
**Feeling empowered and supported by the online world**		
	**Empowerment**	21, 22, 23, 24, 25, 26, 27, 29, 30, 32, 33, 36, 37, 38, 39, 40, 41, 42, 43 **[19]**
	**Sense of community**	23, 24, 25, 26, 27, 28, 29, 30, 33, 35, 36, 37, 38, 39, 40, 43 **[16]**
**Concerns and perceived risks of the online world**		
	**Prior unsatisfactory experiences of seeking information and support online**	21, 22, 25, 28, 29, 31, 32, 34, 35, 36, 27, 38, 39, 41, 42, 43 **[16]**
	**Steered away by health professionals**	22, 26, 29, 32, 34, 38 **[6]**
	**Fear of what could find out online**	22, 25, 28, 29, 34, 38, 39, 42 **[8]**

Table 4. Overview of analytical and descriptive themes across the presenting papers.

For peer review only - http://bmjopen.bmj.com/site/about/guidelines.xhtml

How do parents develop personal strategies for obtaining and appraising information and support in the online world?

Parents were found to have **personal preferences** for certain types of websites and their content (22, 24, 27, 28, 29, 32, 33, 34, 35, 36, 37, 38, 39, 40, 41, 42, 43). Parents reported often favouring certain websites over others, which included official health information sites, charity websites, research sites, online discussion forums, and social media sites. From within the different types of websites, parents also expressed informational preferences including medically moderated content, personalised advice, and content dedicated to the condition without advertisements and other irrelevances. Parents also expressed functional preferences for online resources which included closed/private communities, live chat rooms, and notifications for new content. *"For me, this is a funnel. This group funnels you. People can get quick answers, but if you want more in-depth information, we always do the link to a website because it has way more in-depth information than what you can get in the group online."* (29; Gastro-oesophageal reflux disease).

Parents also described a variety of **searching methods** which enabled them to obtain online information and support (22, 24, 29, 31, 32, 34, 35, 37, 38, 39, 42, 43)**.** Some visited the Internet with a specific question in mind, others reported typing in keywords that they had heard during their medical consultations, and others posted questions in a variety of online resources and combined the information with advice received offline. *"The same question on WhatsApp, Facebook and at the school" (32; various conditions including cystic fibrosis, metabolic conditions, other disabilities).*

Parents also reported **appraising the trustworthiness and credibility** of online resources, which influenced whether they decided to use the online resource (22, 24, 27, 28, 29, 31, 32, 34, 35, 36, 37, 38, 43). Some had reported more specific tactics such as matching up information from a variety of websites, checking the affiliation/source of the website or the background of individual posters, the possible sales and marketing intentions behind the websites, comparing the advice with existing knowledge, whereas others implied that the reliability of online resources had been at least somewhat on their mind. *"[The website] that came up first, they've probably got advertisements in there… everything is paid, so I am a bit sus on that"* (22; Subacute conditions).

Parents' **perceived needs for information and support** also appeared to influence whether and how they searched for information and support online (21, 22, 23, 26, 27, 28, 29, 33, 34, 35, 36, 37, 38, 39, 40, 41, 42, 43). This involved the present stage and severity of their child's condition, such as a recent diagnosis or a change in symptoms, and an unsatisfying recent medical consultation. *"Five minutes after we got the diagnosis… we got on the computer. I didn't know what else to do." (42; Genetic conditions).*

Finally, parents also expressed **past positive experiences with the online world**, and how these often encouraged continuous use (21, 22, 24, 27, 28, 29, 31, 35, 36, 37, 38, 39, 40, 41, 42, 43). Experiences included the Internet's convenient and accessible nature for obtaining

For peer review only - http://bmjopen.bmj.com/site/about/guidelines.xhtml

advice any time of the day, through a variety of mediums, for exploring sensitive topics and within the protection of anonymity, and general successes of obtaining desired information. *"You know when you post even though it's like 5:00 in the morning, you've got other people that are also awake and even though it is 5:00 in the morning, they respond as well within a timely manner." (29; Gastro-oesophageal reflux disease)*.

How and why do parents feel empowered and supported by the online world?

Parents across nearly all papers emphasised feeling **empowered** through accessing and using information and support obtained online (21, 22, 23, 24, 25, 26, 27, 29, 30, 32, 33, 36, 37, 38, 39, 40, 41, 42, 43). Sense of empowerment was reported to derive from parents' development of condition-related knowledge including information about diagnosis, prognosis, triggers, and treatments. Parents often reported that their increase in medical knowledge helped them to feel in more control of managing their children's condition and to cope with stress and during uncertain times such as changes in symptoms and treatments. Accessing information online was also reported to empower parents in subsequent medical consultations, helping them to feel more equipped to understand what their health professionals were telling them, to structure questions, and to recommend plans for their children's treatments. Parents also reported feeling empowered through being able to help others by sharing their own experiences online about managing their children's conditions, such as with treatments, consultations, and coping methods. *"I think it allows you to then make a decision on how serious something might be or whether you need to take action straight away or if it's something that you can potentially wait up and see what happens" (22; subacute conditions).*

The **sense of community** from using social media sites, discussion forums and blogs for peer-support was also emphasised (23, 24, 25, 26, 27, 28, 29, 30, 33, 35, 36, 37, 38, 39, 40, 43). Connecting with other parents with similar experiences online was described as comforting, and often reduced their loneliness and sorrow. The importance of building trust and relationships in online communities was also reported, which enabled parents to gain more advice from one another, to share common language, to be treated as equals, and to constantly be there to support one another. Parents also demonstrated that exchanging experiences online helped them to feel more reassured and validated about their situations. Also, parents expressed how online information and support that derives from others' experiences is invaluable, which often lacks in health professionals' advice. *"...I'm so happy that I found this page. For me to share & read about that there are other parents in my shoes. It breaks my heart watching my little girl try so hard to walk & play with other kids... Her condition has really taken a toll on me. Physically, mentally, & emotionally. I cry a lot. I'm just relieved there's parents on here that I can open up to." (23; cerebral palsy)*

How and why do parents develop concerns and perceive potential risks from seeking information and support online?

For peer review only - http://bmjopen.bmj.com/site/about/guidelines.xhtml

Whilst not as common as the reports of empowerment and support, some parents reported the negative side to information and support online. Some parents reported that **prior unsatisfactory experiences of seeking information and support online** had discouraged them from using online resources (21, 22, 25, 28, 29, 31, 32, 34, 35, 36, 27, 38, 39, 41, 42, 43). Previous experiences included not being able to find their desired information and support, being overwhelmed by the volume of content online, feeling that the Internet does not replace real people, and a lack of digital literacy skills and experience. *"I choose not to [go online] because there are so many diverse opinions out there. There's so much garbage out there." (38; Cancer).*

Parents also reported experiences of being **steered away by health professionals** from using the Internet for information and support (22, 26, 29, 32, 34, 38). This was predominantly in papers that focused more on life-limiting conditions (e.g. needing surgery in first year of life, gastro esophageal reflux disease, cerebral palsy, neurological conditions cancer). *"[Physician B] was very adamant, "Don't you dare touch that Internet, do not look at it, do not— you listen to what I say, I'm the boss, and this is the way it's going to run… I just wanted the definition … I just wanted to know what the words meant…."* (34; Conditions requiring neurosurgery).

Finally, some parents reported that the **fear of what they could find out online** prevented them from using online resources for information and support (22, 25, 28, 29, 34, 38, 39, 42). Some reported having read distressing stories in the past, and others simply did not want to risk uncovering distressing information that they could relate to. These papers also focused more on life-limiting conditions (cancer, type 1 diabetes, rare genetic conditions, neurological). *"I was careful with going online because I think the internet can be a really scary place. We got into a couple of those forums that tend to be sort of doom and gloom and focus on all the negative things. It's all the people that are struggling the most that seem to post in some of those forums."* (28; type 1 diabetes)

Discussion

Summary of findings

This systematic review and thematic synthesis revealed that parents develop a variety of strategies to obtain and appraise information and support online in the context of caring for childhood long-term conditions. These processes were based on their personal preferences, their search methods, the outcome of their appraisal of trustworthiness, the degree of their perceived need for information and support or previous positive experiences online. In most cases, parents reported going on to experience the benefits of accessing information and support online. This included feeling empowered through improving their medical knowledge, which helped them feel more in control in subsequent medical consultations and through being able to help similar others by sharing their own experiences. The sense of community gained through connecting with others in a similar situation online, building relationships and trust, and feeling reassured and validated also highlighted the peer-support that is available from online support communities especially. However, some

For peer review only - http://bmjopen.bmj.com/site/about/guidelines.xhtml

parents also emphasised the concerns and perceived risks of obtaining and using information and support online. These often stemmed from prior unsuccessful online experiences, having been told to avoid the Internet by health professionals, and due to the fear of the distressing stories that they might come across online.

Findings in context of existing research

The findings from this study are largely consistent with other reviews that have explored peoples' experiences with health information online (5-6), although these reviews had not focused specifically on the online experiences of parents of children with long-term physical conditions. However our review uncovered more about the initial processes through which parents develop search strategies to obtain and evaluate health information and support online and the motivations behind these, which lead to the subsequent positive and negative experiences identified. Our review also drew more attention to the negative experiences that parents experience online compared to these reviews (5-6), which could be due to the additional challenges of caring for a child rather than self-caring.

Collating and synthesising the experiences of parents of children with a variety of long-term conditions provided additional insights into parents' experiences with the online world. Drawing on the different experiences discussed suggested that the potential harms of the online world were most apparent for parents of children with life-limiting conditions, or whose conditions were unstable (e.g. cerebral palsy, cancer, type 1 diabetes, rare genetic conditions, gastro-oesophageal reflux disease). Concerns and perceived risks were not found in the papers focusing on eczema, asthma and other allergies, and other disabilities. This suggests that parents of children with life-limiting or unstable conditions use information and support online with more caution.

This review was also able to further identify that the types of information and support sought by parents differed according to the stage and severity of their children's conditions. The need for condition-related information was again more apparent across the papers that focussed on the life-limiting or unstable conditions (e.g. cancers, cystic fibrosis, rare genetic conditions, type 1 diabetes, eczema, asthma, diabetes) whereas the need for peer-support was apparent across all stages and severities (life-limiting and uncertain to stable and well-managed). This suggests that parents' need for condition-specific information comes first and is not needed long-term, whereas the need for peer-support is continuous.

Strengths and Limitations

This is the first systematic review to synthesise the existing qualitative literature exploring parents' experiences of seeking health information and support online for long-term physical childhood conditions, providing new insights and developing the research field in this area.

A limitation to this study was that some of the included studies for synthesis also explored parents' online experiences for conditions that are not long-term and physical (e.g. acute conditions, disabilities of learning, language and cognition, mental health conditions, and health promotion in childhood), within the same paper. Some of the findings in these papers

For peer review only - http://bmjopen.bmj.com/site/about/guidelines.xhtml

were not separated according to different conditions, therefore this review fails to identify possible differences between the online experiences of parents of children with long-term physical conditions, and other types of conditions, although this was not ever an aim of the study. As well as this, included studies typically presented limited demographic data so it was not possible to explore how such factors may affect parents' experiences in the online world' We also excluded quantitative studies which could have been used to support the qualitative findings, and did not search the grey literature which could have excluded some relevant studies. Finally, we analysed only the results sections of each paper, meaning that broader contextual factors that may have arisen in the discussion sections were not included although the discussion sections were read and checked in case.

Implications and future research

Improved understanding of parents' experiences of seeking health information and support online for long-term physical childhood conditions may help health professionals treating these families. The findings from this study could be used to inform health professionals about parents' experiences online: such as what they want from information and support online, why they choose online resources, what they're influenced by, and the types of information and support that they access online.

Parents' preferences and perceived benefits of online resources could be useful for future studies designing online resources for parents of children with long-term conditions. Specific examples that this study identified were online resources that contain medically moderated content, reminders to use online resources, frequently active members to reply to posts, easy to use, and information tailored to the child's condition and its severity. A future systematic review could collate which online resources for certain conditions are evidence-based and meet the needs of parents, so that health professionals can sign-post patients to such online resources.

Conclusion

This systematic review and synthesis of qualitative literature comprehensively explored parents' experiences with health information online for long-term physical childhood conditions. This highlights the support and empowerment that parents receive from online resources, but also their perceptions of potential risks and the processes by which they attempt to establish trustworthiness of sources. These findings could help health professionals to understand parents' perspectives about information and support online, and potentially help them identify robust resources that also meet patients' needs.

For peer review only - http://bmjopen.bmj.com/site/about/guidelines.xhtml

References

1. Eiser C. Chronic childhood disease: An introduction to psychological theory and research. Cambridge University Press; 1990 Sep 27.27.

2. Smith J, Cheater F, Bekker H. Parents' experiences of living with a child with a long-term condition: a rapid structured review of the literature. Health Expectations. 2015 Aug;18(4):452-74.

3. Wainstein BK, Sterling-Levis K, Baker SA, Taitz J, Brydon M. Use of the Internet by parents of paediatric patients. Journal of paediatrics and child health. 2006 Sep;42(9):528-32.

4. Plantin L, Daneback K. Parenthood, information and support on the internet. A literature review of research on parents and professionals online. BMC family practice. 2009 Dec;10(1):1-2.

5. Ziebland SU, Wyke S. Health and illness in a connected world: how might sharing experiences on the internet affect people's health?. The Milbank Quarterly. 2012 Jun;90(2):219-49.

6. Allen C, Vassilev I, Kennedy A, Rogers A. Long-term condition self-management support in online communities: a meta-synthesis of qualitative papers. Journal of medical Internet research. 2016;18(3):e61.

7. Hamm MP, Shulhan J, Williams G, Milne A, Scott SD, Hartling L. A systematic review of the use and effectiveness of social media in child health. BMC pediatrics. 2014 Dec 1;14(1):138.

8. Kinnane NA, Milne DJ. The role of the Internet in supporting and informing carers of people with cancer: a literature review. Supportive Care in Cancer. 2010 Sep 1;18(9):1123-36.

9. Dissemination CF. Systematic reviews: CRD's guidance for undertaking reviews in healthcare. York: University of York NHS Centre for Reviews & Dissemination. 2009.

10. Tong A, Flemming K, McInnes E, Oliver S, Craig J. Enhancing transparency in reporting the synthesis of qualitative research: ENTREQ. BMC medical research methodology. 2012 Dec;12(1):181.

11. Department of Health and Social Care. Policy Paper: 2010 to 2015 government policy: long term health conditions. 2015. Accessed on 22 June 2020 from: https://www.gov.uk/government/publications/2010-to-2015-government-policy-long-term-health-conditions/2010-to-2015-government-policy-long-term-health-conditions#issue

12. Gorecki CA, Brown JM, Briggs M, Nixon J. Evaluation of five search strategies in retrieving qualitative patient-reported electronic data on the impact of pressure ulcers on quality of life. Journal of advanced nursing. 2010 Mar;66(3):645-52.

13. Critical Skills Appraisal Programme. CASP Qualitative Checklist. 2018.

14. Thomas J, Harden A. Methods for the thematic synthesis of qualitative research in systematic reviews. BMC medical research methodology. 2008 Dec 1;8(1):45.

For peer review only - http://bmjopen.bmj.com/site/about/guidelines.xhtml

15. Santer M, Ring N, Yardley L, Geraghty AW, Wyke S. Treatment non-adherence in pediatric long-term medical conditions: systematic review and synthesis of qualitative studies of caregivers' views. BMC pediatrics. 2014 Dec 1;14(1):63.

16. Morton RL, Tong A, Howard K, Snelling P, Webster AC. The views of patients and carers in treatment decision making for chronic kidney disease: systematic review and thematic synthesis of qualitative studies. BmJ. 2010 Jan 19;340:c112.

17. Joseph-Williams N, Elwyn G, Edwards A. Knowledge is not power for patients: a systematic review and thematic synthesis of patient-reported barriers and facilitators to shared decision making. Patient education and counseling. 2014 Mar 1;94(3):291-309.

18. Hefferon K, Grealy M, Mutrie N. Post-traumatic growth and life threatening physical illness: A systematic review of the qualitative literature. British journal of health psychology. 2009 May;14(2):343-78.

19. Pocock M, Trivedi D, Wills W, Bunn F, Magnusson J. Parental perceptions regarding healthy behaviours for preventing overweight and obesity in young children: a systematic review of qualitative studies. Obesity reviews. 2010 May;11(5):338-53.

20. Bridges J, Flatley M, Meyer J. Older people's and relatives' experiences in acute care settings: Systematic review and synthesis of qualitative studies. International journal of nursing studies. 2010 Jan 1;47(1):89-107.

21. Alsem MW, Verhoef M, Braakman J, van Meeteren KM, Siebes RC, Jongmans MJ, Visser-Meily JM, Ketelaar M, WWW, roadmap study group. Parental empowerment in paediatric rehabilitation: Exploring the role of a digital tool to help parents prepare for consultation with a physician. Child: Care, Health and Development. 2019 Sep;45(5):623-36.

22. Benedicta B, Caldwell PH, Scott KM. How parents use, search for and appraise online health information on their child's medical condition: A pilot study. Journal of Paediatrics and Child Health. 2020 Feb;56(2):252-8.

23. Canty MJ, Breitbart S, Siegel L, Fehlings D, Milo-Manson G, Alotaibi NM, Ibrahim GM. The role of social media in selective dorsal rhizotomy for children: information sharing and social support. Child's Nervous System. 2019 Nov 1;35(11):2179-85.

24. Gage-Bouchard EA, LaValley S, Devonish JA. Deciphering the Signal From the Noise: Caregivers' Information Appraisal and Credibility Assessment of Cancer-Related Information Exchanged on Social Networking Sites. Cancer Control. 2019 Apr 20;26(1):1073274819841609.

25. Aston J, Wilson KA, Terry DR. The treatment-related experiences of parents, children and young people with regular prescribed medication. International journal of clinical pharmacy. 2019 Feb 15;41(1):113-21.

26. Hinton L, Locock L, Long AM, Knight M. What can make things better for parents when babies need abdominal surgery in their first year of life? A qualitative interview study in the UK. BMJ open. 2018 Jun 1;8(6):e020921.

For peer review only - http://bmjopen.bmj.com/site/about/guidelines.xhtml

27. Holtz BE, Mitchell KM, Nuttall AK, Cotten SR, Hershey DD, Dunneback JK, Wood MA. Using user-feedback to develop a website: MyT1DHope, for parents of children with T1D. Health communication. 2020 Feb 23;35(3):281-8.

28. Perez L, Romo LK, Bell T. Communicatively Exploring Uncertainty Management of Parents of Children with Type 1 Diabetes. Health Communication. 2019 Jul 29;34(9):949-57.

29. Wright JV. Parents Caring, Sharing, and Learning Together Online: An Exploratory Look at Informal Learning via a Health-Related Support Group in Facebook. Arizona State University; 2018.

30. Rehman S, Lyons K, McEwen R, Sellen K. Motives for sharing illness experiences on Twitter: conversations of parents with children diagnosed with cancer. Information, Communication & Society. 2018 Apr 3;21(4):578-93.

31. Tracey D, Johnston C, Papps FA, Mahmic S. How do parents acquire information to support their child with a disability and navigate individualised funding schemes?. Journal of Research in Special Educational Needs. 2018 Jan;18(1):25-35.

32. Alsem MW, Ausems F, Verhoef M, Jongmans MJ, Meily-Visser JM, Ketelaar M. Information seeking by parents of children with physical disabilities: An exploratory qualitative study. Research in developmental disabilities. 2017 Jan 1;60:125-34.

33. Kirk S, Milnes L. An exploration of how young people and parents use online support in the context of living with cystic fibrosis. Health Expectations. 2016 Apr;19(2):309-21.

34. Vogel TK, Kleib M, Davidson SJ, Scott SD. Parental Evaluation of a Nurse Practitioner-Developed Pediatric Neurosurgery Website. JMIR research protocols. 2016;5(2):e55.

35. Santer M, Muller I, Yardley L, Burgess H, Ersser SJ, Lewis-Jones S, Little P. 'You don't know which bits to believe': qualitative study exploring carers' experiences of seeking information on the internet about childhood eczema. BMJ open. 2015 Apr 1;5(4):e006339.

36. Cargnin Pimentel ED, dos Santos Luz G, Marisa Pelloso S, Barros Carvalho MD. Using the internet to exchange information and experience on cystic fibrosis. Investigación y Educación en Enfermería. 2013 Dec;31(3):457-63.

37. Nordfeldt S, Ängarne-Lindberg T, Nordwall M, Krevers B. Parents of adolescents with type 1 diabetes-their views on information and communication needs and internet use. A qualitative study. PloS one. 2013 Apr 23;8(4):e62096.

38. Gage EA, Panagakis C. The devil you know: parents seeking information online for paediatric cancer. Sociology of health & illness. 2012 Mar;34(3):444-58.

39. Gundersen T. 'One wants to know what a chromosome is': the internet as a coping resource when adjusting to life parenting a child with a rare genetic disorder. Sociology of health & illness. 2011 Jan;33(1):81-95.

For peer review only - http://bmjopen.bmj.com/site/about/guidelines.xhtml

40. Stewart M, Letourneau N, Masuda JR, Anderson S, McGhan S. Online solutions to support needs and preferences of parents of children with asthma and allergies. Journal of Family Nursing. 2011 Aug;17(3):357-79.

41. Nordfeldt S, Hanberger L, Berterö C. Patient and parent views on a Web 2.0 Diabetes Portal—the management tool, the generator, and the gatekeeper: qualitative study. Journal of medical Internet research. 2010;12(2):e17.

42. Roche MI, Skinner D. How parents search, interpret, and evaluate genetic information obtained from the internet. Journal of genetic counseling. 2009 Apr 1;18(2):119-29.

43. Nettleton S, Burrows R, O'Malley L, Watt I. Health E-types' Information. Communication & Society. 2004;7(4):531-53.

For peer review only

For peer review only - http://bmjopen.bmj.com/site/about/guidelines.xhtml

Contributors

Funding This study was part of a PhD studentship funded by the National Institute for Health Research (NIHR) School for Primary Care Research. The views expressed are those of the authors and not necessarily those of the NIHR or the Department of Health and Social Care.

Competing interests None.

Data sharing Unavailable.

For peer review only

For peer review only - http://bmjopen.bmj.com/site/about/guidelines.xhtml

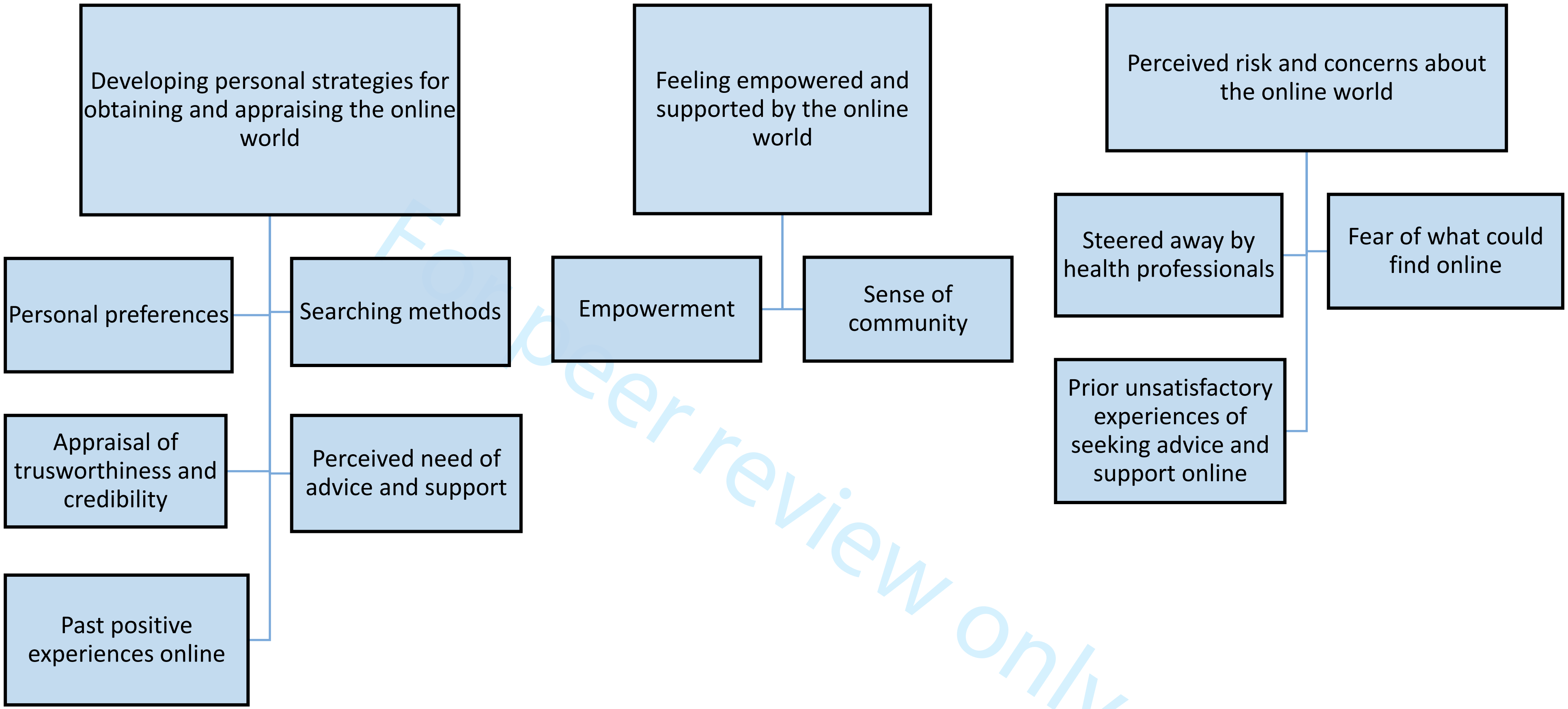

Figure 2. Illustration of the developed analytical themes and descriptive themes.

Bethan Treadgold supplementary file 2

Reporting checklist for systematic review and meta-analysis.

Based on the PRISMA guidelines.

Instructions to authors

Complete this checklist by entering the page numbers from your manuscript where readers will find each of the items listed below.

Your article may not currently address all the items on the checklist. Please modify your text to include the missing information. If you are certain that an item does not apply, please write "n/a" and provide a short explanation.

Upload your completed checklist as an extra file when you submit to a journal.

In your methods section, say that you used the PRISMAreporting guidelines, and cite them as:

Moher D, Liberati A, Tetzlaff J, Altman DG, The PRISMA Group. Preferred Reporting Items for Systematic Reviews and Meta-Analyses: The PRISMA Statement

		Reporting Item	Page Number
**Title**			
	#1	Identify the report as a systematic review, meta-analysis, or both.	1

For peer review only

Abstract

Structured summary	#2	Provide a structured summary including, as applicable: background; objectives; data sources; study eligibility criteria, participants, and interventions; study appraisal and synthesis methods; results; limitations; conclusions and implications of key findings; systematic review registration number	2

Introduction

Rationale	#3	Describe the rationale for the review in the context of what is already known.	4
Objectives	#4	Provide an explicit statement of questions being addressed with reference to participants, interventions, comparisons, outcomes, and study design (PICOS).	4

Methods

Protocol and registration	#5	Indicate if a review protocol exists, if and where it can be accessed (e.g., Web address) and, if available, provide registration information including the registration number.	4
Eligibility criteria	#6	Specify study characteristics (e.g., PICOS, length of follow-up) and report characteristics (e.g., years considered, language, publication status) used as criteria for eligibility, giving rational	4
Information sources	#7	Describe all information sources in the search (e.g., databases with dates of coverage, contact with study authors to identify additional studies) and date last searched.	5

For peer review only

Search	#8	Present full electronic search strategy for at least one database, including any limits used, such that it could be repeated.	6
Study selection	#9	State the process for selecting studies (i.e., for screening, for determining eligibility, for inclusion in the systematic review, and, if applicable, for inclusion in the meta-analysis).	8
Data collection process	#10	Describe the method of data extraction from reports (e.g., piloted forms, independently by two reviewers) and any processes for obtaining and confirming data from investigators.	8
Data items	#11	List and define all variables for which data were sought (e.g., PICOS, funding sources), and any assumptions and simplifications made.	n/a
Risk of bias in individual studies	#12	Describe methods used for assessing risk of bias in individual studies (including specification of whether this was done at the study or outcome level, or both), and how this information is to be used in any data synthesis.	n/a
Summary measures	#13	State the principal summary measures (e.g., risk ratio, difference in means).	n/a
Planned methods of analysis	#14	Describe the methods of handling data and combining results of studies, if done, including measures of consistency (e.g., I2) for each meta-analysis.	8

For peer review only

Risk of bias across studies	#15	Specify any assessment of risk of bias that may affect the cumulative evidence (e.g., publication bias, selective reporting within studies).	n/a
Additional analyses	#16	Describe methods of additional analyses (e.g., sensitivity or subgroup analyses, meta-regression), if done, indicating which were pre-specified.	n/a
**Results**			
Study selection	#17	Give numbers of studies screened, assessed for eligibility, and included in the review, with reasons for exclusions at each stage, ideally with a flow diagram.	8
Study characteristics	#18	For each study, present characteristics for which data were extracted (e.g., study size, PICOS, follow-up period) and provide the citation.	8
Risk of bias within studies	#19	Present data on risk of bias of each study and, if available, any outcome-level assessment (see Item 12).	n/a
Results of individual studies	#20	For all outcomes considered (benefits and harms), present, for each study: (a) simple summary data for each intervention group and (b) effect estimates and confidence intervals, ideally with a forest plot.	n/a
Synthesis of results	#21	Present the main results of the review. If meta-analyses are done, include for each, confidence intervals and measures of consistency.	14

For peer review only

Risk of bias across studies	#22	Present results of any assessment of risk of bias across studies (see Item 15).	n/a
Additional analysis	#23	Give results of additional analyses, if done (e.g., sensitivity or subgroup analyses, meta-regression [see Item 16]).	n/a
**Discussion**			
Summary of Evidence	#24	Summarize the main findings, including the strength of evidence for each main outcome; consider their relevance to key groups (e.g., health care providers, users, and policy makers	18
Limitations	#25	Discuss limitations at study and outcome level (e.g., risk of bias), and at review level (e.g., incomplete retrieval of identified research, reporting bias).	19
Conclusions	#26	Provide a general interpretation of the results in the context of other evidence, and implications for future research.	20
**Funding**			
Funding	#27	Describe sources of funding or other support (e.g., supply of data) for the systematic review; role of funders for the systematic review.	25

The PRISMA checklist is distributed under the terms of the Creative Commons Attribution License CC-BY. This checklist was completed on 25. June 2020 using https://www.goodreports.org/, a tool made by the EQUATOR Network in collaboration with Penelope.ai

For peer review only

[illegible]

Bethan Treadgold supplementary file 1

Enhancing transparency in reporting the synthesis of qualitative research: the ENTREQ statement

From: Tong, A., Flemming, K., McInnes, E. *et al.* Enhancing transparency in reporting the synthesis of qualitative research: ENTREQ. *BMC Med Res Methodol* **12,** 181 (2012). https://doi.org/10.1186/1471-2288-12-181

No	Item	Guide and description	Page no.
**1**	Aim	State the research question the synthesis addresses.	4
**2**	Synthesis methodology	Identify the synthesis methodology or theoretical framework which underpins the synthesis, and describe the rationale for choice of methodology *(e.g. meta-ethnography, thematic synthesis, critical interpretive synthesis, grounded theory synthesis, realist synthesis, meta-aggregation, meta-study, framework synthesis).*	8
**3**	Approach to searching	Indicate whether the search was pre-planned (*comprehensive search strategies to seek all available studies)* or iterative (*to seek all available concepts until they theoretical saturation is achieved).*	5
**4**	Inclusion criteria	Specify the inclusion/exclusion criteria *(e.g. in terms of population, language, year limits, type of publication, study type).*	4
**5**	Data sources	Describe the information sources used (e.g. *electronic databases (MEDLINE, EMBASE, CINAHL, psycINFO, Econlit), grey literature databases (digital thesis, policy reports), relevant organisational websites, experts, information specialists, generic web searches (Google Scholar) hand searching, reference lists)* and when the searches conducted; provide the rationale for using the data sources.	5
**6**	Electronic Search strategy	Describe the literature search *(e.g. provide electronic search strategies with population terms, clinical or health topic terms, experiential or social phenomena related terms, filters for qualitative research, and search limits).*	6
**7**	Study screening methods	Describe the process of study screening and sifting *(e.g. title, abstract and full text review, number of independent reviewers who screened studies).*	8
**8**	Study characteristics	Present the characteristics of the included studies *(e.g. year of publication, country, population, number of participants, data collection, methodology, analysis, research questions).*	8

**9**	Study selection results	Identify the number of studies screened and provide reasons for study exclusion *(e,g, for comprehensive searching, provide numbers of studies screened and reasons for exclusion indicated in a figure/flowchart; for iterative searching describe reasons for study exclusion and inclusion based on modifications t the research question and/or contribution to theory development).*	8
**10**	Rationale for appraisal	Describe the rationale and approach used to appraise the included studies or selected findings *(e.g. assessment of conduct (validity and robustness), assessment of reporting (transparency), assessment of content and utility of the findings).*	8
**11**	Appraisal items	State the tools, frameworks and criteria used to appraise the studies or selected findings *(e.g. Existing tools: CASP, QARI, COREQ, Mays and Pope* [25]*; reviewer developed tools; describe the domains assessed: research team, study design, data analysis and interpretations, reporting).*	8
**12**	Appraisal process	Indicate whether the appraisal was conducted independently by more than one reviewer and if consensus was required.	8
**13**	Appraisal results	Present results of the quality assessment and indicate which articles, if any, were weighted/excluded based on the assessment and give the rationale.	14
**14**	Data extraction	Indicate which sections of the primary studies were analysed and how were the data extracted from the primary studies? *(e.g. all text under the headings "results /conclusions" were extracted electronically and entered into a computer software).*	8
**15**	Software	State the computer software used, if any.	8
**16**	Number of reviewers	Identify who was involved in coding and analysis.	8
**17**	Coding	Describe the process for coding of data *(e.g. line by line coding to search for concepts).*	8
**18**	Study comparison	Describe how were comparisons made within and across studies *(e.g. subsequent studies were coded into pre-existing concepts, and new concepts were created when deemed necessary).*	8
**19**	Derivation of themes	Explain whether the process of deriving the themes or constructs was inductive or deductive.	8
**20**	Quotations	Provide quotations from the primary studies to illustrate themes/constructs, and identify whether the quotations were participant quotations of the author's interpretation.	16
**21**	Synthesis output	Present rich, compelling and useful results that go beyond a summary of the primary studies (e.g. *new interpretation, models of evidence, conceptual models, analytical framework, development of a new theory or construct).*	16

For peer review only

Parents and carers' experiences of seeking health information and support online for long-term physical childhood conditions: A systematic review and thematic synthesis of qualitative research

Journal:	*BMJ Open*
Manuscript ID	bmjopen-2020-042139.R1
Article Type:	Original research
Date Submitted by the Author:	29-Sep-2020
Complete List of Authors:	Treadgold, Bethan; University of Southampton Faculty of Medicine, Primary Care, Population Sciences and Medical Education<br>Teasdale, Emma; University of Southampton Faculty of Medicine, Primary Care, Population Sciences and Medical Education<br>Muller, Ingrid; University of Southampton Faculty of Medicine, Primary Care, Population Sciences and Medical Education<br>Roberts, Amanda; University of Nottingham Faculty of Medicine and Health Sciences, Centre of Evidence-Based Dermatology<br>Coulson, Neil; University of Nottingham Faculty of Medicine and Health Sciences, Division of Rehabilitation, Ageing and Wellbeing<br>Santer, Miriam; University of Southampton Faculty of Medicine, Primary Care, Population Sciences and Medical Education
**Primary Subject Heading**:	Qualitative research
Secondary Subject Heading:	Paediatrics
Keywords:	PAEDIATRICS, QUALITATIVE RESEARCH, World Wide Web technology < BIOTECHNOLOGY & BIOINFORMATICS

SCHOLARONE™
Manuscripts

I, the Submitting Author has the right to grant and does grant on behalf of all authors of the Work (as defined in the below author licence), an exclusive licence and/or a non-exclusive licence for contributions from authors who are: i) UK Crown employees; ii) where BMJ has agreed a CC-BY licence shall apply, and/or iii) in accordance with the terms applicable for US Federal Government officers or employees acting as part of their official duties; on a worldwide, perpetual, irrevocable, royalty-free basis to BMJ Publishing Group Ltd ("BMJ") its licensees and where the relevant Journal is co-owned by BMJ to the co-owners of the Journal, to publish the Work in this journal and any other BMJ products and to exploit all rights, as set out in our licence.

The Submitting Author accepts and understands that any supply made under these terms is made by BMJ to the Submitting Author unless you are acting as an employee on behalf of your employer or a postgraduate student of an affiliated institution which is paying any applicable article publishing charge ("APC") for Open Access articles. Where the Submitting Author wishes to make the Work available on an Open Access basis (and intends to pay the relevant APC), the terms of reuse of such Open Access shall be governed by a Creative Commons licence – details of these licences and which Creative Commons licence will apply to this Work are set out in our licence referred to above.

Other than as permitted in any relevant BMJ Author's Self Archiving Policies, I confirm this Work has not been accepted for publication elsewhere, is not being considered for publication elsewhere and does not duplicate material already published. I confirm all authors consent to publication of this Work and authorise the granting of this licence.

For peer review only

Parents and carers' experiences of seeking health information and support online for long-term physical childhood conditions:

A systematic review and thematic synthesis of qualitative research

Bethan M. Treadgold[1], Emma J. Teasdale[1], Ingrid Muller[1], Amanda Roberts[2], Neil S. Coulson[3], Miriam Santer[1]

[1] School of Primary Care, Population Health and Medical Education, University of Southampton, UK

[2] Nottingham Support Group for Carers of Children with Eczema, Nottingham, UK

[3] School of Medicine, Division of Rehabilitation, Ageing and Wellbeing, University of Nottingham, UK

Word count: 3609 (Including everything from Introduction to Conclusion except for tables and figures. In-text quotations and table/figure labels are included.)

Corresponding author:

Bethan M. Treadgold

School of Primary Care, Population Health and Medical Education, University of Southampton, UK

b.m.treadgold@southampton.ac.uk

For peer review only - http://bmjopen.bmj.com/site/about/guidelines.xhtml

Abstract

Objective To systematically review and synthesise qualitative research exploring parents/carers' experiences of seeking online information and support for long-term physical childhood conditions.

Design Systematic review and thematic synthesis of qualitative research.

Data sources Medline, CINAHL, Embase, PsycINFO, and The International Bibliography of the Social Sciences were searched from inception to September 2019. We used thematic synthesis to analyse findings.

Eligibility criteria Primary research papers presenting qualitative data collection and analysis, focusing on parents/carers' experiences of seeking health information and support from online resources for long-term physical childhood health conditions. No language restrictions were placed.

Results 23 studies from seven countries met inclusion criteria and were included in the synthesis. Included studies presented data collected through interviews/focus groups with 559 parents/carers; free-text surveys and essays with 26 parents/carers; and 2407 messages from online support groups. Parents/carers developed a variety of strategies to obtain information and support online, based on personal preferences, appraisal of trustworthiness, perceived needs, and previous experiences online. Many parents sought the benefits of online information and support, which included reassurance and validation from online communities, and feeling they had greater knowledge about their children's conditions. Some concerns and perceived risks were discussed, which often stemmed from prior unsatisfactory experiences of seeking information and support online, consultations with health professionals, and seeing distressing stories online.

Conclusion Most parents/carers were successful in obtaining information and support online. Many continued to share experiences with other parents/carers online. The need for information was particularly apparent early after diagnosis of the condition, whereas the need for peer-support continued. The potential concerns and perceived risks with information and support online were especially apparent amongst parents/carers of children with life-limiting long-term conditions. Findings may be useful for health professionals to facilitate discussions regarding use of online resources, and researchers designing online health resources for parents/carers.

PROSPERO registration number The protocol for this study was registered with PROSPERO in May 2018 [CRD42018096009].

For peer review only - http://bmjopen.bmj.com/site/about/guidelines.xhtml

Strengths and limitations of this study

- Comprehensive study summarising and providing new insights into the online experiences of parents/carers of children with long-term physical conditions
- Useful for health professionals and researchers interacting with this population
- Excludes the online experiences of parents/carers of children with acute, mental health, or developmental conditions, and otherwise healthy children

For peer review only

For peer review only - http://bmjopen.bmj.com/site/about/guidelines.xhtml

Background

Caring for a child with a long-term condition can be challenging for parents and carers, requiring ongoing management with regular treatment use, and often complicated, time-consuming and emotionally-laden everyday routines (1). Seeking to maintain treatment and care regimes, dealing with social and financial constraints, whilst balancing the needs of the whole family increase these challenges (2). For the remainder of the paper, parents and carers are collectively referred to as ‘parents’.

As a source of health information, the Internet is convenient, instant and can help enhance knowledge and understanding of a condition (3). As a source of support, the Internet also enables parents to exchange stories with other parents with similar experiences. This commonly occurs through asynchronous discussion platforms (4), although synchronous (real-time) discussions through online platforms are also increasingly popular. Reviews of qualitative research have explored how the Internet influences peoples’ health experiences (5, 6). Experiences include obtaining health information to understand illness, redressing offline information and knowledge deficits, discussing experiences with health services, building connections with others, feeling supported and validated, releasing emotions and offline frustrations, and learning self-management behaviours from others. Other reviews of qualitative research surrounding this area have explored the views of physicians regarding social media interventions to support child health (7), and experiences with health information online for carers of adults living with cancer (8). However, no systematic review has been conducted that synthesises the qualitative research on the online experiences of parents of children with long-term physical conditions.

Aim

This review aims to explore parents’ experiences of seeking information and support online for long-term physical childhood conditions.

Method

This study follows The Centre for Reviews and Disseminations guidance for undertaking reviews in health care (9), and the ENTREQ guidelines for reporting the synthesis of qualitative research (10). The protocol for this study was registered with PROSPERO in May 2018 [CRD 2016018096009].

Eligibility criteria

We sought studies that explored parents’ experiences of using the Internet for information and support about long-term physical childhood condition(s) (Table 1). To be eligible for inclusion, studies had to present qualitative data collection and analysis methods, and qualitative data. Studies that used a mixed methodology were included if they comprised a qualitative component substantive enough to perform a qualitative analysis.

	Inclusion criteria	Exclusion criteria

For peer review only - http://bmjopen.bmj.com/site/about/guidelines.xhtml

**Setting**	• Online health information and support resources (e.g. websites, forums, social media pages, blogs)	• Primarily offline health information and support resources (e.g. family, friends, books, leaflets)
**Design**	• Qualitative data collection methods (or mixed-methods studies involving qualitative data) (e.g. interviews), qualitative data analysis methods (e.g. thematic analysis) and presented qualitative data (e.g. themes, narratives and quotations)<br>• Primary research (e.g. full research papers, feasibility studies and pilot studies)	• Did not present the qualitative study criteria<br>• Not primary research (e.g. reviews, protocols and commentaries)
**Population**	• Parents and carers of children living with long-term physical health conditions	• Not parents or carers (e.g. family members without caring responsibility for the child, health professionals and teachers)
**Condition**	• Long-term physical health conditions<br>- Definition: A long-term or chronic physical health condition is a condition that requires ongoing management which cannot currently be cured but can be controlled with regular adherence to treatment (11)	• Acute conditions (e.g. common cold)<br>• Mental health conditions (e.g. depression)<br>• Developmental disorders of learning, language and cognition (e.g. Attention Deficit Hyperactivity Disorder)<br>• Healthy populations or health-promoting related studies (e.g. diet, physical activity, obesity)
**Outcome**	• Parents and carers' experiences	• Not parents or carers' experiences (e.g. patterns of Internet usage derived from quantitative studies)

Table 1. Eligibility criteria.

Search strategy

Five electronic databases were searched (Medline, CINAHL, Embase, PsycINFO, and The International Bibliography of the Social Sciences) from the earliest date possible to September 2019. The Cochrane Library of Systematic Reviews and PROSPERO were also searched to check for any ongoing similar reviews. A comprehensive search strategy was developed with a medical librarian from the University of Southampton for each database (Table 2). Search terms seeking to identify only qualitative research were initially included in search strategies, but we found that they did not improve the search results so were subsequently removed. Others have similarly found subject-specific search strategies rather than methodology-based strategies to be more effective in identifying all relevant studies reporting qualitative findings (12).

For peer review only - http://bmjopen.bmj.com/site/about/guidelines.xhtml

Database	Internet search terms	Parent/carer search terms	Use of online health information search terms	Paediatric search terms
**MEDLINE**	1. exp Internet/<br>2. forum*.mp.<br>3. facebook.mp.<br>4. social media.mp.<br>5. blog*.mp.<br>6. Internet.mp.<br>7. (web or online).mp.	1. parents/ or fathers/ or mothers/ or single parent/<br>2. Caregivers/<br>3. Legal Guardians/<br>4. (parent or parents).mp.<br>5. caregiver*.mp.<br>6. carer*.mp.<br>7. guardian*.mp.	1. Help-seeking Behaviour/<br>2. health information.mp.<br>3. Self-Help Groups/<br>4. self help.mp.<br>5. exp Health Education/<br>6. patient information.mp.<br>7. medical information.mp.<br>8. health information.mp.	1. Child Health/<br>2. Child/<br>3. Pediatrics/<br>4. (child or children or paediatric* or pediatric*).mp.
**CINAHL**	1. SU Internet<br>2. forum<br>3. facebook<br>4. social media<br>5. blog*<br>6. Internet	1. SU (parent* OR father* OR mother* OR single parent*)<br>2. SU caregivers<br>3. SU legal guardians<br>4. (parent OR parents)<br>5. caregiver*<br>6. carer*<br>7. guardian*	1. SU help seeking behaviour<br>2. health information<br>3. SU self help groups<br>4. self help<br>5. SU health education<br>6. patient information<br>7. medical information<br>8. health information	1. SU child health<br>2. SU child<br>3. SU paediatrics<br>4. (child OR children OR paediatric* OR pediatric*)
**Embase**	1. exp Internet/<br>2. forum*.mp.<br>3. facebook.mp.<br>4. social media.mp.<br>5. blog*.mp.<br>6. Internet.mp.<br>7. (web or online).mp	1. parents/ or fathers/ or mothers/ or single parent/<br>2. Caregivers/<br>3. Legal Guardians/<br>4. (parent or parents).mp.<br>5. caregiver*.mp.<br>6. carer*.mp.<br>7. guardian*.mp	1. Help-seeking Behaviour/<br>2. health information.mp.<br>3. Self-Help Groups/<br>4. self help.mp.<br>5. exp Health Education/<br>6. patient information.mp.<br>7. medical information.mp.<br>8. health information.mp.	1. Child Health/<br>2. Child/<br>3. Pediatrics/<br>4. (child or children or paediatric* or pediatric*).mp.
**PsycINFO**	1. SU Internet<br>2. forum<br>3. facebook<br>4. social media<br>5. blog*<br>6. Internet	1. SU (parent* OR father* OR mother* OR single parent*)<br>2. SU caregivers<br>3. SU legal guardians<br>4. (parent OR parents)<br>5. caregiver*<br>6. carer*<br>7. guardian*	1. SU help seeking behaviour<br>2. health information<br>3. SU self help groups<br>4. self help<br>5. SU health education<br>6. patient information<br>7. medical information<br>8. health information	1. SU child health<br>2. SU child<br>3. SU paediatrics<br>4. (child OR children OR paediatric* OR pediatric*)

For peer review only - http://bmjopen.bmj.com/site/about/guidelines.xhtml

**International Bibliography of Social Science**	1. Ab (Internet* OR forum* OR facebook OR social media OR blog* OR web* OR online)	1. Ab (parent* OR father* OR mother* OR caregiver* OR guardian* OR carer*)	1. Ab (help seeking behaviour* OR health information OR self help group* OR self help* OR health education OR patient information OR medical information)	1. Ab (child health OR child* OR paediatric* OR pediatric*)

Table 2. Search terms and syntax for each database.

For peer review only

For peer review only - http://bmjopen.bmj.com/site/about/guidelines.xhtml

Study screening methods

One author (BT) screened the titles and abstracts of all identified papers against the inclusion criteria. Duplicates of papers, and those that failed to meet the inclusion criteria were eliminated during this stage. A second author (ET) screened 20% of the titles and abstracts to check for consistency. Full papers for the remaining identified studies were retrieved and assessed for eligibility (see Figure 1).

Quality appraisal and data extraction

One author (BT) extracted information from the included studies and tabulated it. Assessors (BT, IM, MS, ET) independently assessed the quality of the included papers, using the Critical Appraisal Skills Programme (CASP) quality assessment tool for qualitative research (13). Any discrepancies were discussed with the research team.

Thematic Synthesis

We followed thematic synthesis for the analysis of the included studies (14), as this method has been successfully used for synthesising qualitative research in systematic reviews in health (15-20). Thematic synthesis consists of three stages. In stages 1 and 2 we explored and described the data. One author (BT) read and re-read included papers, then inductively coded each line of the studies' findings according to their content and meaning to develop an initial coding frame. Participants' original data excerpts, as well as the authors' descriptions of findings, were coded in the latest version of NVivo. The coding frame was then discussed and refined within the research team (BT, ET, IM, AR, NC, MS). In stage 3 we aimed to 'go beyond' individual study findings through developing descriptive themes initially and, subsequently, generating analytical themes.

Patient and Public Involvement

We involved an experienced public contributor with experience of eczema (AR) in the aim, design, analysis and write-up stages of the review. AR has a great deal of experience with online support groups, and has often highlighted that the online environment can be very supportive whilst also potentially bewildering. The contributor's experiences and preferences therefore helped shape the aim and design of the review: to explore parents' experiences of seeking information and support online for long-term physical childhood conditions.

Findings

Study selection

The database searches resulted in 2981 records after excluding duplicates. A final 23 (21-43) studies met all inclusion criteria and were included for thematic synthesis (Figure 1).

Study characteristics

All studies were published in English between 2004 and 2019, with the majority of studies (n = 14) published since 2016. 11 studies were conducted in the Americas (USA (6), Canada (4), Brazil (1)), 10 studies were conducted in Europe (UK (5), Sweden (2), The Netherlands (2),

For peer review only - http://bmjopen.bmj.com/site/about/guidelines.xhtml

and Norway (1)), and 2 studies were conducted in Australia. The 23 studies presented the experiences of 559 parents captured through interviews and focus groups, 26 parents through free-text surveys and essays, and analyses of 2407 messages that had been posted by parents in online support groups (e.g. online forums, social media sites and blogs), though it should be noted that not all studies provided sample size information (Table 3).

For peer review only

For peer review only - http://bmjopen.bmj.com/site/about/guidelines.xhtml

Authors of study (Country) (ref.)	Aim(s)	Method of data collection (sample size)	Method of data analysis (as written by the authors)	Condition(s)
**Alsem et al., 2019 (The Netherlands) (21)**	To explore parental and physicians' experiences with the WWW-roadmap, as well as differences between parents who had used the WWW-roadmap to prepare for a consultation with their rehabilitation physician and parents who had not, in terms of empowerment and self-efficacy, parental and professional satisfaction with the consultation and family centredness of care.	Semi-structured interviews (sample size not stated)	Theoretical thematic analysis	Disabilities: Cerebral palsy , Neuromuscular, Metabolic disorder, Genetic disorder, Spina bifida, Various syndromes (e.g., Down's), Movement disorder, Unknown, Other * Included conditions that did not meet inclusion criteria.
**Benedicta et al., 2019 (Australia) (22)**	To understand how parents search for and appraise the relevance and credibility of online health information regarding their child, and how they use this information in order to help them make better health decisions for their child.	Think aloud task of searching online health info and semi-structured interviews (22)	Observation checklist for the think aloud task; inductive thematic analysis for the interviews	'Subacute' conditions * Potentially included conditions that did not meet inclusion criteria. Definition of 'subacute' not provided.
**Canty et al., 2019 (Canada) (23)**	To qualitatively and quantitatively assess the use of social media as an information-sharing and support-seeking tool by patients and caregivers**.**	Analysis of social media postings (sample size not stated)	Modified thematic analysis	Cerebral palsy
**Gage-Bouchard et al., 2019 (USA) (24)**	To examine the reasons paediatric cancer caregivers engage with cancer-related information on SNS and how caregivers appraise and assess the credibility of cancer-related information they encounter on this platform.	Semi-structured interviews (40)	Thematic analysis	Cancer
**Aston et al., 2018 (UK) (25)**	To explore the treatment-related experiences when children and young people take regular prescribed medication.	Semi-structured interviews (23 parents)	Thematic analysis	In-patients taking two or more prescribed medications concurrently at home, prior to admission, for 6 weeks or longer. Names of conditions not provided

For peer review only - http://bmjopen.bmj.com/site/about/guidelines.xhtml

				* Possibly included conditions that did not meet inclusion criteria.
**Hinton et al., 2018 (UK) (26)**	To explore parents' experiences and perspectives of having a baby who needs early abdominal surgery; identify the questions and problems that matter to parents during and after their pregnancy and infant's surgery and identify the long-term impact on parents and families.	Interviews (starting unstructured then semi-structured) (44)	Interpretative thematic analysis using a modified grounded theory approach, with constant comparison and exploration of deviant cases	Infants who needed abdominal surgery in the first year of life<br>* Included conditions that did not meet inclusion criteria.
**Holtz et al., 2018 (USA) (27)**	To develop a website resource, as an initial step in a larger intervention, for parents with a child with Type 1 Diabetes.	Focus groups (3 parents), interviews (3)	Iterative thematic analysis	Type 1 Diabetes
**Perez et al., 2018 (USA) (28)**	Stated as RQs: RQ1: What types of uncertainty do parents of a child with Type 1 Diabetes report experiencing?; RQ2: How do parents manage this uncertainty?	In-depth interviews (29)	Thematic analysis	Type 1 Diabetes
**Wright 2018 (USA) (29)**	To qualitatively explore the informal learning experiences of members of an online social media group hosted by Facebook.	Semi-structured interviews (25) and analyses of 604 postings (original and comments) created by members of the online social media	Several Grounded Theory methodologies	Gastro-oesophageal reflux disease
**Rehman et al., 2018 (Canada) (30)**	To establish the dominant themes of tweets posted by parents of children diagnosed with cancer and to situate the themes within a theoretical framework that describes the phenomenon of information sharing within a non-work context.	Analysis of the content of 1700 tweets from the Twitter accounts of 15 recruited participants	A deductive subjective interpretation	Cancer
**Tracey et al., 2018 (Australia) (31)**	To explore how parents acquire information to enhance their understanding of their child's disability and individualised funding schemes.	Focus groups (56 parents, 13 groups)	A a two stage, mixed method sequential approach which followed an initial survey	'disability'<br>* Possibly included conditions that did not meet inclusion criteria.
**Alsem et al., 2017 (The Netherlands) (32)**	To explore the experiences of Dutch parents of children with disabilities as regards their information needs, and to describe the process	Semi-structured interviews (15)	Open coding thematic analysis	Cerebral palsy, GMFCSa 2–5, rare disorders (e.g. genetic syndromes and metabolic and mitochondrial diseases)

For peer review only - http://bmjopen.bmj.com/site/about/guidelines.xhtml

	of seeking and evaluating information and the different sources of information.			* Possibly included conditions that did not meet inclusion criteria.
**Kirk & Milnes, 2016 (UK) (33)**	To explore how online peer support is used by young people and parents to support self-care in relation to cystic fibrosis.	Analysis of discussion group postings (103)	Inductive grounded theory	Cystic fibrosis
**Vogel et al., 2016 (Canada) (34)**	To explore parental usage of the National Kidney Foundation website, track visitor behaviour, evaluate usability and design, establish ways to improve user experience, and identify ways to redesign the website.	Mixed methods: Google analytic usage reports and focus groups (4)	Inductive content analysis and constant comparative approach	A condition requiring neurosurgery
**Santer et al., 2015 (UK) (35)**	To explore parents and carers' experiences of searching for information about childhood eczema on the Internet.	Semi-structured interviews (31)	Content analysis and constant comparative approach	Eczema
**Pimentel et al., 2013 (Brazil) (36)**	To describe the experience of patients with cystic fibrosis and their families using the Internet as a means for information, interaction and exchange of experiences with this disease.	Questionnaire (7)	Content analysis	Cystic fibrosis
**Nordfeldt et al., 2013 (Sweden) (37)**	To explore parents' views on their information-seeking, Internet use, and social networking online, and to identify implications for the future development of Internet use in this respect.	Focus groups (27)	Inductive qualitative content analysis	Type 1 Diabetes
**Gage & Panagakis, 2012 (USA) (38)**	To examine how parents think about, evaluate, access and use the Internet to seek information related to their child's cancer.	Mixed methods: Survey and structured interviews (41)	Grounded theory	Cancer
**Gundersen, 2011 (Norway) (39)**	To explore how the Internet can function as a resource that parents can draw on to cope with their situation.	Semi-structured interviews (10)	Not a specified method but looks like thematic analysis with comparative approach based on theorist	Rare genetic disorders that entail developmental and/or physical problems (e.g. allergies and epilepsy, and two had severe physical and learning problems)<br>* Included conditions that did not meet inclusion criteria.

For peer review only - http://bmjopen.bmj.com/site/about/guidelines.xhtml

**Stewart et al., 2011 (Canada) (40)**	To gather parents' preferences of support to design an innovative online peer support and test it.	Semi-structured interviews (44)	Deductive thematic content analysis	Asthma and allergies
**Nordfeldt et al., 2010 (Sweden) (41)**	To explore patients' and parents' attitudes toward a local Web 2.0 portal tailored to young patients with type 1 diabetes and their parents.	Essays (19)	Content analysis	Type 1 Diabetes
**Roche & Skinner, 2009 (USA) (42)**	To describe how parents of a child referred for genetic services searched the Internet for information, summarise how they interpreted and evaluated the information they obtained, and identify barriers that they encountered.	Semi-structured interviews (100)	Constant comparative method	Genetic
**Nettleton et al., 2004 (UK) (43)**	To explore parents and children's experiences of using the Internet for chronic childhood conditions.	Qualitative interviews (69)	Strategic comparisons approach'	Eczema, asthma, diabetes

Table 3. Summaries of the final 23 papers for thematic synthesis.

For peer review only - http://bmjopen.bmj.com/site/about/guidelines.xhtml

Quality of studies

The quality assessment of the included studies revealed that all studies met most quality indicators. All studies had clear statements of the aims of the research, qualitative methodology was appropriate in all studies and was collected in a way that addressed the aims. We did not exclude any studies based on quality of reporting.

Synthesis of results

The papers included in this review broadly focused on how parents of children with long-term physical conditions use online resources for health information and support, and what they gain (or not) from using online resources. By synthesising the papers' findings, we interpreted three main analytical themes: 1) Developing personal strategies for obtaining and appraising the online world; 2) Feeling supported and informed by the online world; 3) Perceived risk and concerns about the online world.

Figure 2 illustrates the analytical themes and descriptive themes, and table 4 lists all analytical and descriptive themes, along with references to the relevant papers.

For peer review only

For peer review only - http://bmjopen.bmj.com/site/about/guidelines.xhtml

Analytical theme	Descriptive theme	Paper references [total number]
**Developing personal strategies for obtaining and appraising the online world**		
	**Personal preferences**	22, 24, 27, 28, 29, 32, 33, 34, 35, 36, 37, 38, 39, 40, 41, 42, 43 **[17]**
	**Searching methods**	22, 24, 29, 31, 32, 34, 35, 37, 38, 39, 42, 43 **[12]**
	**Appraisal of trustworthiness and credibility**	22, 24, 27, 28, 29, 31, 32, 34, 35, 36, 37, 38, 43 **[13]**
	**Perceived need of information and support**	21, 22, 23, 26, 27, 28, 29, 33, 34, 35, 36, 37, 38, 39, 40, 41, 42, 43 **[18]**
	**Past positive experiences online**	21, 22, 24, 27, 28, 29, 31, 35, 36, 37, 38, 39, 40, 41, 42, 43 **[16]**
**Feeling supported and informed by the online world**		
	**Sense of community**	23, 24, 25, 26, 27, 28, 29, 30, 33, 35, 36, 37, 38, 39, 40, 43 **[16]**
	**Feeling informed**	21, 22, 23, 24, 25, 26, 27, 29, 30, 32, 33, 36, 37, 38, 39, 40, 41, 42, 43 **[19]**
**Concerns and perceived risks of the online world**		
	**Prior unsatisfactory experiences of seeking information and support online**	21, 22, 25, 28, 29, 31, 32, 34, 35, 36, 27, 38, 39, 41, 42, 43 **[16]**
	**Steered away by health professionals**	22, 26, 29, 32, 34, 38 **[6]**
	**Fear of what could find out online**	22, 25, 28, 29, 34, 38, 39, 42 **[8]**

Table 4. Overview of analytical and descriptive themes across the presenting papers.

For peer review only - http://bmjopen.bmj.com/site/about/guidelines.xhtml

How do parents develop personal strategies for obtaining and appraising information and support in the online world?

Parents were found to have **personal preferences** for certain types of websites and their content (22, 24, 27, 28, 29, 32, 33, 34, 35, 36, 37, 38, 39, 40, 41, 42, 43). Parents reported often favouring certain websites over others, which included official health information sites, charity websites, research sites, online discussion forums, and social media sites. From within the different types of websites, parents also expressed informational preferences including medically moderated content, personalised advice, and content dedicated to the condition without advertisements and other irrelevances. Parents also expressed functional preferences for online resources which included closed/private communities, live chat rooms, and notifications for new content. *"For me, this is a funnel. This group funnels you. People can get quick answers, but if you want more in-depth information, we always do the link to a website because it has way more in-depth information than what you can get in the group online."* (29; Gastro-oesophageal reflux disease, Interview and online forum study).

Parents also described a variety of **searching methods** which enabled them to obtain online information and support (22, 24, 29, 31, 32, 34, 35, 37, 38, 39, 42, 43)**.** Some visited the Internet with a specific question in mind, others reported typing in keywords that they had heard during their medical consultations, and others posted questions in a variety of online resources and combined the information with advice received offline. *"The same question on WhatsApp, Facebook and at the school"* (32; Various conditions including cystic fibrosis, metabolic conditions, other disabilities, Interview study).

Parents also reported **appraising the trustworthiness and credibility** of online resources, which influenced whether they decided to use the online resource (22, 24, 27, 28, 29, 31, 32, 34, 35, 36, 37, 38, 43). Some had reported more specific tactics such as matching up information from a variety of websites, checking the affiliation/source of the website or the background of individual posters, the possible sales and marketing intentions behind the websites, and comparing the advice with existing knowledge, whereas others implied that the reliability of online resources had been at least somewhat on their mind. *"[The website] that came up first, they've probably got advertisements in there… everything is paid, so I am a bit sus on that"* (22; Subacute conditions, Think aloud and interview study).

Parents' **perceived needs for information and support** also appeared to influence whether and how they searched for information and support online (21, 22, 23, 26, 27, 28, 29, 33, 34, 35, 36, 37, 38, 39, 40, 41, 42, 43). This involved the present stage and severity of their child's condition, such as a recent diagnosis or a change in symptoms, and an unsatisfying recent medical consultation. *"Five minutes after we got the diagnosis… we got on the computer. I didn't know what else to do."* (42; Genetic conditions, Interview study).

Finally, parents also expressed **past positive experiences with the online world**, and how these often encouraged continuous use (21, 22, 24, 27, 28, 29, 31, 35, 36, 37, 38, 39, 40, 41, 42, 43). Experiences included the Internet's convenient and accessible nature for obtaining

For peer review only - http://bmjopen.bmj.com/site/about/guidelines.xhtml

advice any time of the day, through a variety of mediums, for exploring sensitive topics and within the protection of anonymity, and general successes of obtaining desired information. *"You know when you post even though it's like 5:00 in the morning, you've got other people that are also awake and even though it is 5:00 in the morning, they respond as well within a timely manner."* (29; Gastro-oesophageal reflux disease, Interview and online forum study).

How and why do parents feel supported and informed by the online world?

The **sense of community** from using social media sites, discussion forums and blogs for peer-support was also emphasised (23, 24, 25, 26, 27, 28, 29, 30, 33, 35, 36, 37, 38, 39, 40, 43). Connecting with other parents with similar experiences online was described as comforting, and often reduced their loneliness and sorrow. The importance of building trust and relationships in online communities was also reported, which enabled parents to gain more advice from one another, to share common language, to be treated as equals, and to constantly be there to support one another. Parents also demonstrated that exchanging experiences online helped them to feel more reassured and validated about their situations. Also, parents expressed how online information and support that derives from others' experiences is invaluable, which often lacks in health professionals' advice. *"...I'm so happy that I found this page. For me to share & read about that there are other parents in my shoes. It breaks my heart watching my little girl try so hard to walk & play with other kids... Her condition has really taken a toll on me. Physically, mentally, & emotionally. I cry a lot. I'm just relieved there's parents on here that I can open up to."* (23; Cerebral palsy, Online forum study)

Parents across nearly all papers emphasised feeling **informed** through accessing and using information and support obtained online (21, 22, 23, 24, 25, 26, 27, 29, 30, 32, 33, 36, 37, 38, 39, 40, 41, 42, 43). The sense of feeling better informed was reported to derive from parents' development of condition-related knowledge including information about diagnosis, prognosis, triggers, and treatments. Parents often reported that their increase in medical knowledge helped them to feel in more control of managing their children's condition, and to cope with stress and during uncertain times such as changes in symptoms and treatments. Accessing information online was also reported to facilitate parents in subsequent medical consultations, through helping them to feel more equipped to understand what their health professionals were telling them, to structure questions, and to recommend plans for their children's treatments. Parents also reported feeling good about sharing their own experiences online about managing their children's conditions, such as with treatments, consultations, and coping methods. *"I think it allows you to then make a decision on how serious something might be or whether you need to take action straight away or if it's something that you can potentially wait up and see what happens"* (22; Subacute conditions, Think aloud and interview study).

How and why do parents develop concerns and perceive potential risks from seeking information and support online?

For peer review only - http://bmjopen.bmj.com/site/about/guidelines.xhtml

Whilst less common than positive accounts of feeling supported and informed, some parents reported the negative side to information and support online. Some parents reported that **prior unsatisfactory experiences of seeking information and support online** had discouraged them from using online resources (21, 22, 25, 28, 29, 31, 32, 34, 35, 36, 27, 38, 39, 41, 42, 43). Previous experiences included not being able to find their desired information and support, being overwhelmed by the volume of content online, feeling that the Internet does not replace real people, and a lack of digital literacy skills and experience. *"I choose not to [go online] because there are so many diverse opinions out there. There's so much garbage out there."* (38; Cancer, Survey and interview study).

Parents also reported experiences of being **steered away by health professionals** from using the Internet for information and support (22, 26, 29, 32, 34, 38). This was predominantly in papers that focused more on life-limiting conditions (e.g. needing surgery in first year of life, gastro esophageal reflux disease, cerebral palsy, neurological, cancer). *"[Physician B] was very adamant, "Don't you dare touch that Internet, do not look at it, do not— you listen to what I say, I'm the boss, and this is the way it's going to run… I just wanted the definition … I just wanted to know what the words meant…."* (34; Conditions requiring neurosurgery, Focus group study).

Finally, some parents reported that the **fear of what they could find out online** prevented them from using online resources for information and support (22, 25, 28, 29, 34, 38, 39, 42). Some reported having read distressing stories in the past, and others simply did not want to risk uncovering distressing information that they could relate to. These papers also focused more on life-limiting conditions (cancer, type 1 diabetes, rare genetic conditions, neurological). *"I was careful with going online because I think the internet can be a really scary place. We got into a couple of those forums that tend to be sort of doom and gloom and focus on all the negative things. It's all the people that are struggling the most that seem to post in some of those forums."* (28; Type 1 diabetes, Interview study).

Discussion

Summary of findings

This systematic review and thematic synthesis revealed that parents develop a variety of strategies to obtain and appraise information and support online in the context of caring for long-term physical childhood conditions. These processes were based on their personal preferences, their search methods, the outcome of their appraisal of trustworthiness, the degree of their perceived need for information and support, and previous positive experiences online. In most cases, parents reported going on to experience the benefits of accessing information and support online. This included the sense of community gained through connecting with others in a similar situation online, building relationships and trust, and feeling reassured and validated, which was available from online support communities especially. Parents also reported feeling better informed about their children's conditions, which helped them feel more in control in subsequent medical consultations and felt good

For peer review only - http://bmjopen.bmj.com/site/about/guidelines.xhtml

about helping similar others by sharing their own experiences. However, some parents also emphasised the concerns and perceived risks of obtaining and using information and support online. These often stemmed from prior unsuccessful online experiences, having been told to avoid the Internet by health professionals, and due to the fear of the distressing stories that they might come across online.

Findings in context of existing research

The findings from this study are largely consistent with other reviews that have explored peoples' experiences with health information online (5-6), although these reviews had not focused specifically on the online experiences of parents of children with long-term physical conditions. However our review uncovered more about the initial processes through which parents develop search strategies to obtain and evaluate health information and support online and the motivations behind these, which lead to the subsequent positive and negative experiences identified. Our review also drew more attention to the negative experiences that parents experience online compared to these reviews (5-6), which could be due to the additional challenges of caring for a child rather than self-caring.

Collating and synthesising the experiences of parents of children with a variety of long-term conditions provided additional insights into parents' experiences with the online world. Drawing on the different experiences discussed suggested that the potential harms of the online world were most apparent for parents of children with life-limiting conditions, or whose conditions were unstable (e.g. cerebral palsy, cancer, type 1 diabetes, rare genetic conditions, gastro-oesophageal reflux disease). Concerns and perceived risks were not found in the papers focusing on eczema, asthma and other allergies, and other disabilities. This suggests that parents of children with life-limiting or unstable conditions use information and support online with more caution.

This review was also able to further identify that the types of information and support sought by parents differed according to the stage and severity of their children's conditions. The need for condition-related information was again more apparent across the papers that focussed on the life-limiting or unstable conditions, whereas the need for peer-support was apparent across all stages and severities (life-limiting and uncertain to stable and well-managed). This suggests that parents' need for condition-specific information comes first and is not needed long-term, whereas the need for peer-support is continuous.

Strengths and Limitations

This is the first systematic review to synthesise the existing qualitative literature exploring parents' experiences of seeking health information and support online for long-term physical childhood conditions, providing new insights and developing the research field in this area.

A limitation to this study was that some of the included studies for synthesis also explored parents' online experiences for conditions that are not long-term and physical (e.g. acute conditions, disabilities of learning, language and cognition, mental health conditions, and health promotion in childhood), within the same paper. Some of the findings in these papers

For peer review only - http://bmjopen.bmj.com/site/about/guidelines.xhtml

were not separated according to different conditions, therefore this review fails to identify possible differences between the online experiences of parents of children with long-term physical conditions, and other types of conditions. However, this was never an aim of the study. Some included papers had limitations in reporting of methodological detail, for instance sample size information. Additionally, included studies typically presented limited demographic data so it was not possible to explore how such factors may affect parents' experiences in the online world. We also excluded quantitative studies which could have been used to support the qualitative findings, and did not search the grey literature which could have excluded some relevant studies. Finally, we analysed only the results sections of each paper, meaning that broader contextual factors that may have arisen in the discussion sections were not included although the discussion sections were read and checked in case.

Implications and future research

Improved understanding of parents' experiences of seeking health information and support online for long-term physical childhood conditions may help health professionals treating these families. The findings from this study could be used to inform health professionals about parents' experiences online: such as what they want from information and support online, why they choose online resources, what they are influenced by, and the types of information and support that they access online.

Parents' preferences and perceived benefits of online resources could be useful for future studies designing online resources for parents of children with long-term conditions. Specific examples that this study identified were online resources that contain medically moderated content, reminders to use online resources, frequently active members to reply to posts, easy to use, and information tailored to the child's condition and its severity. A future systematic review could collate which online resources for certain conditions are evidence-based and meet the needs of parents, so that health professionals can sign-post patients to such online resources.

Conclusion

This systematic review and synthesis of qualitative literature comprehensively explored parents' experiences with health information online for long-term physical childhood conditions. This highlights the support that parents receive from online resources, but also their perceptions of potential risks and the processes by which they attempt to establish trustworthiness of sources. These findings could help health professionals to understand parents' perspectives about information and support online, and potentially help them identify robust resources that also meet patients' needs.

For peer review only - http://bmjopen.bmj.com/site/about/guidelines.xhtml

References

1. Eiser C. Chronic childhood disease: An introduction to psychological theory and research. Cambridge University Press; 1990 Sep 27.27.

2. Smith J, Cheater F, Bekker H. Parents' experiences of living with a child with a long-term condition: a rapid structured review of the literature. Health Expectations. 2015 Aug;18(4):452-74.

3. Wainstein BK, Sterling-Levis K, Baker SA, Taitz J, Brydon M. Use of the Internet by parents of paediatric patients. Journal of paediatrics and child health. 2006 Sep;42(9):528-32.

4. Plantin L, Daneback K. Parenthood, information and support on the internet. A literature review of research on parents and professionals online. BMC family practice. 2009 Dec;10(1):1-2.

5. Ziebland SU, Wyke S. Health and illness in a connected world: how might sharing experiences on the internet affect people's health?. The Milbank Quarterly. 2012 Jun;90(2):219-49.

6. Allen C, Vassilev I, Kennedy A, Rogers A. Long-term condition self-management support in online communities: a meta-synthesis of qualitative papers. Journal of medical Internet research. 2016;18(3):e61.

7. Hamm MP, Shulhan J, Williams G, Milne A, Scott SD, Hartling L. A systematic review of the use and effectiveness of social media in child health. BMC pediatrics. 2014 Dec 1;14(1):138.

8. Kinnane NA, Milne DJ. The role of the Internet in supporting and informing carers of people with cancer: a literature review. Supportive Care in Cancer. 2010 Sep 1;18(9):1123-36.

9. Dissemination CF. Systematic reviews: CRD's guidance for undertaking reviews in healthcare. York: University of York NHS Centre for Reviews & Dissemination. 2009.

10. Tong A, Flemming K, McInnes E, Oliver S, Craig J. Enhancing transparency in reporting the synthesis of qualitative research: ENTREQ. BMC medical research methodology. 2012 Dec;12(1):181.

11. Department of Health and Social Care. Policy Paper: 2010 to 2015 government policy: long term health conditions. 2015. Accessed on 22 June 2020 from: https://www.gov.uk/government/publications/2010-to-2015-government-policy-long-term-health-conditions/2010-to-2015-government-policy-long-term-health-conditions#issue

12. Gorecki CA, Brown JM, Briggs M, Nixon J. Evaluation of five search strategies in retrieving qualitative patient-reported electronic data on the impact of pressure ulcers on quality of life. Journal of advanced nursing. 2010 Mar;66(3):645-52.

13. Critical Skills Appraisal Programme. CASP Qualitative Checklist. 2018.

14. Thomas J, Harden A. Methods for the thematic synthesis of qualitative research in systematic reviews. BMC medical research methodology. 2008 Dec 1;8(1):45.

For peer review only - http://bmjopen.bmj.com/site/about/guidelines.xhtml

15. Santer M, Ring N, Yardley L, Geraghty AW, Wyke S. Treatment non-adherence in pediatric long-term medical conditions: systematic review and synthesis of qualitative studies of caregivers' views. BMC pediatrics. 2014 Dec 1;14(1):63.

16. Morton RL, Tong A, Howard K, Snelling P, Webster AC. The views of patients and carers in treatment decision making for chronic kidney disease: systematic review and thematic synthesis of qualitative studies. BmJ. 2010 Jan 19;340:c112.

17. Joseph-Williams N, Elwyn G, Edwards A. Knowledge is not power for patients: a systematic review and thematic synthesis of patient-reported barriers and facilitators to shared decision making. Patient education and counseling. 2014 Mar 1;94(3):291-309.

18. Hefferon K, Grealy M, Mutrie N. Post-traumatic growth and life threatening physical illness: A systematic review of the qualitative literature. British journal of health psychology. 2009 May;14(2):343-78.

19. Pocock M, Trivedi D, Wills W, Bunn F, Magnusson J. Parental perceptions regarding healthy behaviours for preventing overweight and obesity in young children: a systematic review of qualitative studies. Obesity reviews. 2010 May;11(5):338-53.

20. Bridges J, Flatley M, Meyer J. Older people's and relatives' experiences in acute care settings: Systematic review and synthesis of qualitative studies. International journal of nursing studies. 2010 Jan 1;47(1):89-107.

21. Alsem MW, Verhoef M, Braakman J, van Meeteren KM, Siebes RC, Jongmans MJ, Visser-Meily JM, Ketelaar M, WWW, roadmap study group. Parental empowerment in paediatric rehabilitation: Exploring the role of a digital tool to help parents prepare for consultation with a physician. Child: Care, Health and Development. 2019 Sep;45(5):623-36.

22. Benedicta B, Caldwell PH, Scott KM. How parents use, search for and appraise online health information on their child's medical condition: A pilot study. Journal of Paediatrics and Child Health. 2020 Feb;56(2):252-8.

23. Canty MJ, Breitbart S, Siegel L, Fehlings D, Milo-Manson G, Alotaibi NM, Ibrahim GM. The role of social media in selective dorsal rhizotomy for children: information sharing and social support. Child's Nervous System. 2019 Nov 1;35(11):2179-85.

24. Gage-Bouchard EA, LaValley S, Devonish JA. Deciphering the Signal From the Noise: Caregivers' Information Appraisal and Credibility Assessment of Cancer-Related Information Exchanged on Social Networking Sites. Cancer Control. 2019 Apr 20;26(1):1073274819841609.

25. Aston J, Wilson KA, Terry DR. The treatment-related experiences of parents, children and young people with regular prescribed medication. International journal of clinical pharmacy. 2019 Feb 15;41(1):113-21.

26. Hinton L, Locock L, Long AM, Knight M. What can make things better for parents when babies need abdominal surgery in their first year of life? A qualitative interview study in the UK. BMJ open. 2018 Jun 1;8(6):e020921.

For peer review only - http://bmjopen.bmj.com/site/about/guidelines.xhtml

27. Holtz BE, Mitchell KM, Nuttall AK, Cotten SR, Hershey DD, Dunneback JK, Wood MA. Using user-feedback to develop a website: MyT1DHope, for parents of children with T1D. Health communication. 2020 Feb 23;35(3):281-8.

28. Perez L, Romo LK, Bell T. Communicatively Exploring Uncertainty Management of Parents of Children with Type 1 Diabetes. Health Communication. 2019 Jul 29;34(9):949-57.

29. Wright JV. Parents Caring, Sharing, and Learning Together Online: An Exploratory Look at Informal Learning via a Health-Related Support Group in Facebook. Arizona State University; 2018.

30. Rehman S, Lyons K, McEwen R, Sellen K. Motives for sharing illness experiences on Twitter: conversations of parents with children diagnosed with cancer. Information, Communication & Society. 2018 Apr 3;21(4):578-93.

31. Tracey D, Johnston C, Papps FA, Mahmic S. How do parents acquire information to support their child with a disability and navigate individualised funding schemes?. Journal of Research in Special Educational Needs. 2018 Jan;18(1):25-35.

32. Alsem MW, Ausems F, Verhoef M, Jongmans MJ, Meily-Visser JM, Ketelaar M. Information seeking by parents of children with physical disabilities: An exploratory qualitative study. Research in developmental disabilities. 2017 Jan 1;60:125-34.

33. Kirk S, Milnes L. An exploration of how young people and parents use online support in the context of living with cystic fibrosis. Health Expectations. 2016 Apr;19(2):309-21.

34. Vogel TK, Kleib M, Davidson SJ, Scott SD. Parental Evaluation of a Nurse Practitioner-Developed Pediatric Neurosurgery Website. JMIR research protocols. 2016;5(2):e55.

35. Santer M, Muller I, Yardley L, Burgess H, Ersser SJ, Lewis-Jones S, Little P. 'You don't know which bits to believe': qualitative study exploring carers' experiences of seeking information on the internet about childhood eczema. BMJ open. 2015 Apr 1;5(4):e006339.

36. Cargnin Pimentel ED, dos Santos Luz G, Marisa Pelloso S, Barros Carvalho MD. Using the internet to exchange information and experience on cystic fibrosis. Investigación y Educación en Enfermería. 2013 Dec;31(3):457-63.

37. Nordfeldt S, Ängarne-Lindberg T, Nordwall M, Krevers B. Parents of adolescents with type 1 diabetes-their views on information and communication needs and internet use. A qualitative study. PloS one. 2013 Apr 23;8(4):e62096.

38. Gage EA, Panagakis C. The devil you know: parents seeking information online for paediatric cancer. Sociology of health & illness. 2012 Mar;34(3):444-58.

39. Gundersen T. 'One wants to know what a chromosome is': the internet as a coping resource when adjusting to life parenting a child with a rare genetic disorder. Sociology of health & illness. 2011 Jan;33(1):81-95.

For peer review only - http://bmjopen.bmj.com/site/about/guidelines.xhtml

40. Stewart M, Letourneau N, Masuda JR, Anderson S, McGhan S. Online solutions to support needs and preferences of parents of children with asthma and allergies. Journal of Family Nursing. 2011 Aug;17(3):357-79.

41. Nordfeldt S, Hanberger L, Berterö C. Patient and parent views on a Web 2.0 Diabetes Portal—the management tool, the generator, and the gatekeeper: qualitative study. Journal of medical Internet research. 2010;12(2):e17.

42. Roche MI, Skinner D. How parents search, interpret, and evaluate genetic information obtained from the internet. Journal of genetic counseling. 2009 Apr 1;18(2):119-29.

43. Nettleton S, Burrows R, O'Malley L, Watt I. Health E-types' Information. Communication & Society. 2004;7(4):531-53.

For peer review only

For peer review only - http://bmjopen.bmj.com/site/about/guidelines.xhtml

Figures

Figure 1. PRISMA diagram of systematic search and study.

Figure 2. Illustration of the developed analytical themes and descriptive themes.

For peer review only

For peer review only - http://bmjopen.bmj.com/site/about/guidelines.xhtml

Contributors

Contributorship statement BT developed and refined the protocol, undertook the systematic review, data extraction, quality assessment, carried out the thematic synthesis, and drafted the manuscript. ET refined the protocol, contributed to the eligibility screening process, quality assessment, thematic synthesis, and writing the manuscript. MS and IM refined the protocol, contributed to quality assessment, thematic synthesis, and writing the manuscript. AR and NC refined the protocol, contributed to interpretation of findings, and writing the manuscript. All authors read and approved the final manuscript.

Funding This study was part a PhD studentship funded by the National Institute for Health Research (NIHR) School for Primary Care Research. The views expressed are those of the authors and not necessarily those of the NIHR or the Department of Health and Social Care.

Acknowledgements We would like to thank Paula Sands for her expertise in developing the search strategy for this study.

Competing interests None.

Data sharing No data are available.

For peer review only

For peer review only - http://bmjopen.bmj.com/site/about/guidelines.xhtml

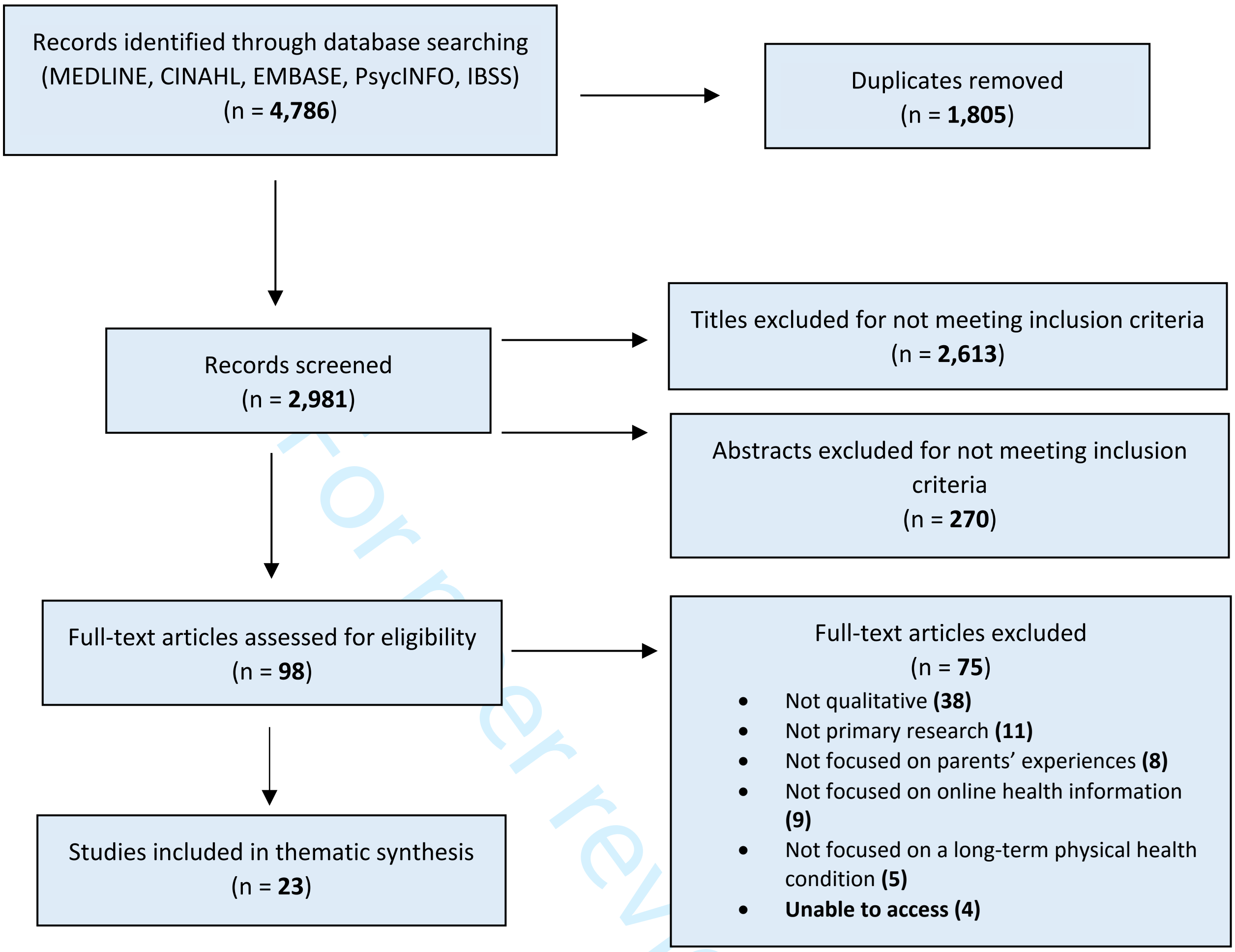

Figure 1. PRISMA diagram of systematic search and study.

For peer review only

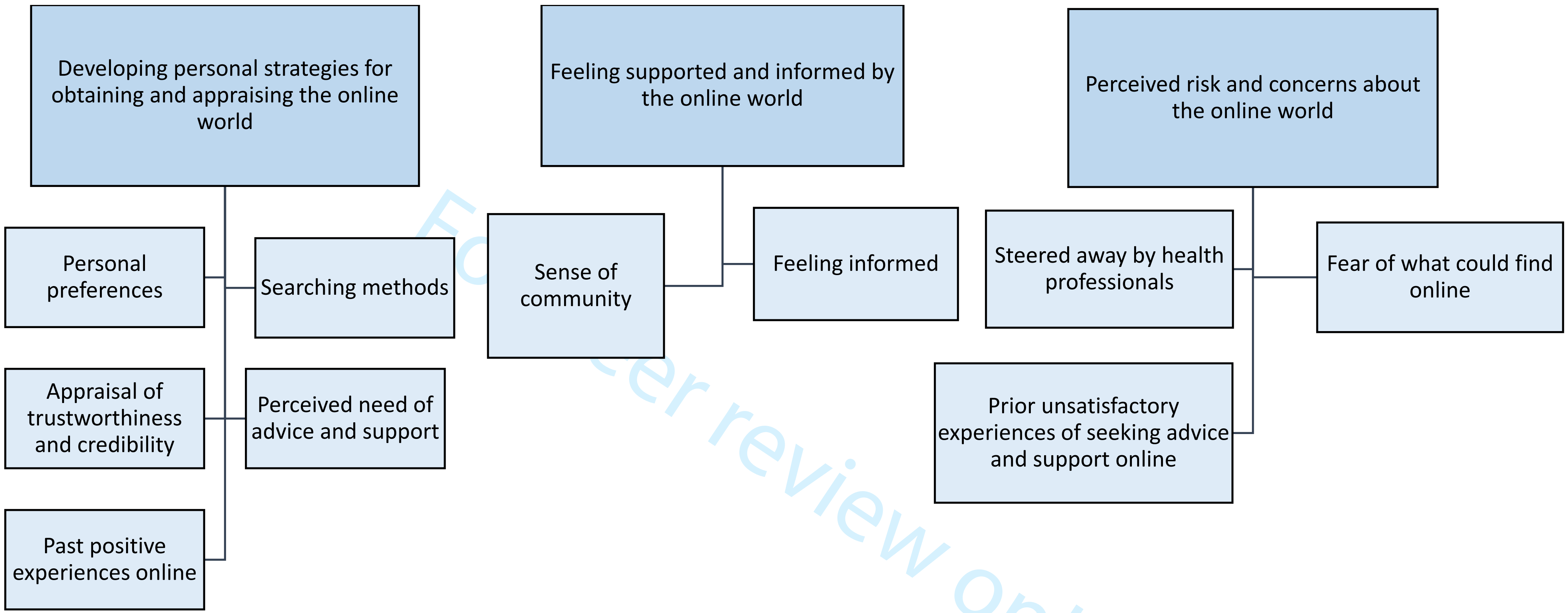

Figure 2. Illustration of the developed analytical themes and descriptive themes.

Reporting checklist for systematic review and meta-analysis.

Based on the PRISMA guidelines.

Instructions to authors

Complete this checklist by entering the page numbers from your manuscript where readers will find each of the items listed below.

Your article may not currently address all the items on the checklist. Please modify your text to include the missing information. If you are certain that an item does not apply, please write "n/a" and provide a short explanation.

Upload your completed checklist as an extra file when you submit to a journal.

In your methods section, say that you used the PRISMAreporting guidelines, and cite them as:

Moher D, Liberati A, Tetzlaff J, Altman DG, The PRISMA Group. Preferred Reporting Items for Systematic Reviews and Meta-Analyses: The PRISMA Statement

		Reporting Item	Page Number
**Title**			
	#1	Identify the report as a systematic review, meta-analysis, or both.	1
**Abstract**			
Structured summary	#2	Provide a structured summary including, as applicable: background; objectives; data sources; study eligibility criteria, participants, and interventions; study appraisal and synthesis methods; results; limitations; conclusions and implications of key findings; systematic review registration number	2
**Introduction**			
Rationale	#3	Describe the rationale for the review in the context of what is already known.	4

For peer review only

Objectives	#4	Provide an explicit statement of questions being addressed with reference to participants, interventions, comparisons, outcomes, and study design (PICOS).	4
**Methods**			
Protocol and registration	#5	Indicate if a review protocol exists, if and where it can be accessed (e.g., Web address) and, if available, provide registration information including the registration number.	4
Eligibility criteria	#6	Specify study characteristics (e.g., PICOS, length of follow-up) and report characteristics (e.g., years considered, language, publication status) used as criteria for eligibility, giving rational	4
Information sources	#7	Describe all information sources in the search (e.g., databases with dates of coverage, contact with study authors to identify additional studies) and date last searched.	5
Search	#8	Present full electronic search strategy for at least one database, including any limits used, such that it could be repeated.	6
Study selection	#9	State the process for selecting studies (i.e., for screening, for determining eligibility, for inclusion in the systematic review, and, if applicable, for inclusion in the meta-analysis).	8
Data collection process	#10	Describe the method of data extraction from reports (e.g., piloted forms, independently by two reviewers) and any processes for obtaining and confirming data from investigators.	8
Data items	#11	List and define all variables for which data were sought (e.g., PICOS, funding sources), and any assumptions and simplifications made.	n/a
Risk of bias in individual studies	#12	Describe methods used for assessing risk of bias in individual studies (including specification of whether this was done at the study or outcome level, or both), and how this information is to be used in any data synthesis.	n/a
Summary measures	#13	State the principal summary measures (e.g., risk ratio, difference in means).	n/a

For peer review only

Planned methods of analysis	#14	Describe the methods of handling data and combining results of studies, if done, including measures of consistency (e.g., I2) for each meta-analysis.	8
Risk of bias across studies	#15	Specify any assessment of risk of bias that may affect the cumulative evidence (e.g., publication bias, selective reporting within studies).	19
Additional analyses	#16	Describe methods of additional analyses (e.g., sensitivity or subgroup analyses, meta-regression), if done, indicating which were pre-specified.	n/a
**Results**			
Study selection	#17	Give numbers of studies screened, assessed for eligibility, and included in the review, with reasons for exclusions at each stage, ideally with a flow diagram.	8
Study characteristics	#18	For each study, present characteristics for which data were extracted (e.g., study size, PICOS, follow-up period) and provide the citation.	8
Risk of bias within studies	#19	Present data on risk of bias of each study and, if available, any outcome-level assessment (see Item 12).	n/a
Results of individual studies	#20	For all outcomes considered (benefits and harms), present, for each study: (a) simple summary data for each intervention group and (b) effect estimates and confidence intervals, ideally with a forest plot.	n/a
Synthesis of results	#21	Present the main results of the review. If meta-analyses are done, include for each, confidence intervals and measures of consistency.	14
Risk of bias across studies	#22	Present results of any assessment of risk of bias across studies (see Item 15).	n/a
Additional analysis	#23	Give results of additional analyses, if done (e.g., sensitivity or subgroup analyses, meta-regression [see Item 16]).	n/a
**Discussion**			
Summary of Evidence	#24	Summarize the main findings, including the strength of evidence for each main outcome; consider their relevance to	18

For peer review only

		key groups (e.g., health care providers, users, and policy makers	
Limitations	#25	Discuss limitations at study and outcome level (e.g., risk of bias), and at review level (e.g., incomplete retrieval of identified research, reporting bias).	19
Conclusions	#26	Provide a general interpretation of the results in the context of other evidence, and implications for future research.	20
**Funding**			
Funding	#27	Describe sources of funding or other support (e.g., supply of data) for the systematic review; role of funders for the systematic review.	26

The PRISMA checklist is distributed under the terms of the Creative Commons Attribution License CC-BY. This checklist was completed on 24. September 2020 using https://www.goodreports.org/, a tool made by the EQUATOR Network in collaboration with Penelope.ai

For peer review only

Bethan Treadgold supplementary file 1

Enhancing transparency in reporting the synthesis of qualitative research: the ENTREQ statement

From: Tong, A., Flemming, K., McInnes, E. *et al.* Enhancing transparency in reporting the synthesis of qualitative research: ENTREQ. *BMC Med Res Methodol* **12,** 181 (2012). https://doi.org/10.1186/1471-2288-12-181

No	Item	Guide and description	Page no.
**1**	Aim	State the research question the synthesis addresses.	4
**2**	Synthesis methodology	Identify the synthesis methodology or theoretical framework which underpins the synthesis, and describe the rationale for choice of methodology *(e.g. meta-ethnography, thematic synthesis, critical interpretive synthesis, grounded theory synthesis, realist synthesis, meta-aggregation, meta-study, framework synthesis).*	8
**3**	Approach to searching	Indicate whether the search was pre-planned (*comprehensive search strategies to seek all available studies)* or iterative (*to seek all available concepts until they theoretical saturation is achieved).*	5
**4**	Inclusion criteria	Specify the inclusion/exclusion criteria *(e.g. in terms of population, language, year limits, type of publication, study type).*	4
**5**	Data sources	Describe the information sources used (e.g. *electronic databases (MEDLINE, EMBASE, CINAHL, psycINFO, Econlit), grey literature databases (digital thesis, policy reports), relevant organisational websites, experts, information specialists, generic web searches (Google Scholar) hand searching, reference lists)* and when the searches conducted; provide the rationale for using the data sources.	5
**6**	Electronic Search strategy	Describe the literature search *(e.g. provide electronic search strategies with population terms, clinical or health topic terms, experiential or social phenomena related terms, filters for qualitative research, and search limits)*.	6
**7**	Study screening methods	Describe the process of study screening and sifting *(e.g. title, abstract and full text review, number of independent reviewers who screened studies).*	8
**8**	Study characteristics	Present the characteristics of the included studies *(e.g. year of publication, country, population, number of participants, data collection, methodology, analysis, research questions).*	8

For peer review only

**9**	Study selection results	Identify the number of studies screened and provide reasons for study exclusion *(e,g, for comprehensive searching, provide numbers of studies screened and reasons for exclusion indicated in a figure/flowchart; for iterative searching describe reasons for study exclusion and inclusion based on modifications t the research question and/or contribution to theory development).*	8
**10**	Rationale for appraisal	Describe the rationale and approach used to appraise the included studies or selected findings *(e.g. assessment of conduct (validity and robustness), assessment of reporting (transparency), assessment of content and utility of the findings).*	8
**11**	Appraisal items	State the tools, frameworks and criteria used to appraise the studies or selected findings *(e.g. Existing tools: CASP, QARI, COREQ, Mays and Pope* [25]*; reviewer developed tools; describe the domains assessed: research team, study design, data analysis and interpretations, reporting).*	8
**12**	Appraisal process	Indicate whether the appraisal was conducted independently by more than one reviewer and if consensus was required.	8
**13**	Appraisal results	Present results of the quality assessment and indicate which articles, if any, were weighted/excluded based on the assessment and give the rationale.	14
**14**	Data extraction	Indicate which sections of the primary studies were analysed and how were the data extracted from the primary studies? *(e.g. all text under the headings "results /conclusions" were extracted electronically and entered into a computer software).*	8
**15**	Software	State the computer software used, if any.	8
**16**	Number of reviewers	Identify who was involved in coding and analysis.	8
**17**	Coding	Describe the process for coding of data *(e.g. line by line coding to search for concepts).*	8
**18**	Study comparison	Describe how were comparisons made within and across studies *(e.g. subsequent studies were coded into pre-existing concepts, and new concepts were created when deemed necessary).*	8
**19**	Derivation of themes	Explain whether the process of deriving the themes or constructs was inductive or deductive.	8
**20**	Quotations	Provide quotations from the primary studies to illustrate themes/constructs, and identify whether the quotations were participant quotations of the author's interpretation.	16
**21**	Synthesis output	Present rich, compelling and useful results that go beyond a summary of the primary studies (e.g. *new interpretation, models of evidence, conceptual models, analytical framework, development of a new theory or construct).*	16

For peer review only

BMJ Open

Parents and carers' experiences of seeking health information and support online for long-term physical childhood conditions: A systematic review and thematic synthesis of qualitative research

Journal:	*BMJ Open*
Manuscript ID	bmjopen-2020-042139.R2
Article Type:	Original research
Date Submitted by the Author:	25-Nov-2020
Complete List of Authors:	Treadgold, Bethan; University of Southampton Faculty of Medicine, Primary Care, Population Sciences and Medical Education<br>Teasdale, Emma; University of Southampton Faculty of Medicine, Primary Care, Population Sciences and Medical Education<br>Muller, Ingrid; University of Southampton Faculty of Medicine, Primary Care, Population Sciences and Medical Education<br>Roberts, Amanda; University of Nottingham Faculty of Medicine and Health Sciences, Centre of Evidence-Based Dermatology<br>Coulson, Neil; University of Nottingham Faculty of Medicine and Health Sciences, Division of Rehabilitation, Ageing and Wellbeing<br>Santer, Miriam; University of Southampton Faculty of Medicine, Primary Care, Population Sciences and Medical Education
**Primary Subject Heading**:	Qualitative research
Secondary Subject Heading:	Paediatrics
Keywords:	PAEDIATRICS, QUALITATIVE RESEARCH, World Wide Web technology < BIOTECHNOLOGY & BIOINFORMATICS

SCHOLARONE™
Manuscripts

I, the Submitting Author has the right to grant and does grant on behalf of all authors of the Work (as defined in the below author licence), an exclusive licence and/or a non-exclusive licence for contributions from authors who are: i) UK Crown employees; ii) where BMJ has agreed a CC-BY licence shall apply, and/or iii) in accordance with the terms applicable for US Federal Government officers or employees acting as part of their official duties; on a worldwide, perpetual, irrevocable, royalty-free basis to BMJ Publishing Group Ltd ("BMJ") its licensees and where the relevant Journal is co-owned by BMJ to the co-owners of the Journal, to publish the Work in this journal and any other BMJ products and to exploit all rights, as set out in our licence.

The Submitting Author accepts and understands that any supply made under these terms is made by BMJ to the Submitting Author unless you are acting as an employee on behalf of your employer or a postgraduate student of an affiliated institution which is paying any applicable article publishing charge ("APC") for Open Access articles. Where the Submitting Author wishes to make the Work available on an Open Access basis (and intends to pay the relevant APC), the terms of reuse of such Open Access shall be governed by a Creative Commons licence – details of these licences and which Creative Commons licence will apply to this Work are set out in our licence referred to above.

Other than as permitted in any relevant BMJ Author's Self Archiving Policies, I confirm this Work has not been accepted for publication elsewhere, is not being considered for publication elsewhere and does not duplicate material already published. I confirm all authors consent to publication of this Work and authorise the granting of this licence.

For peer review only

Parents and carers' experiences of seeking health information and support online for long-term physical childhood conditions:

A systematic review and thematic synthesis of qualitative research

Bethan M. Treadgold[1], Emma J. Teasdale[1], Ingrid Muller[1], Amanda Roberts[2], Neil S. Coulson[3], Miriam Santer[1]

[1] School of Primary Care, Population Health and Medical Education, University of Southampton, UK

[2] Nottingham Support Group for Carers of Children with Eczema, Nottingham, UK

[3] School of Medicine, Division of Rehabilitation, Ageing and Wellbeing, University of Nottingham, UK

For peer review only

Word count: 3612 (Including everything from Introduction to Conclusion except for tables and figures. In-text quotations and table/figure labels are included.)

Corresponding author:

Bethan M. Treadgold

School of Primary Care, Population Health and Medical Education, University of Southampton, UK

b.m.treadgold@southampton.ac.uk

For peer review only - http://bmjopen.bmj.com/site/about/guidelines.xhtml

Abstract

Objective To systematically review and synthesise qualitative research exploring parents/carers' experiences of seeking online information and support for long-term physical childhood conditions.

Design Systematic review and thematic synthesis of qualitative research.

Data sources Medline, CINAHL, Embase, PsycINFO, and The International Bibliography of the Social Sciences were searched from inception to September 2019. We used thematic synthesis to analyse findings.

Eligibility criteria Primary research papers presenting qualitative data collection and analysis, focusing on parents/carers' experiences of seeking health information and support from online resources for long-term physical childhood health conditions. No language restrictions were placed.

Results 23 studies from seven countries met inclusion criteria and were included in the synthesis. Included studies presented data collected through interviews/focus groups with 559 parents/carers; free-text surveys and essays with 26 parents/carers; and 2407 messages from online support groups. Parents/carers developed a variety of strategies to obtain information and support online, based on personal preferences, appraisal of trustworthiness, perceived needs, and previous experiences online. Many parents sought the benefits of online information and support, which included reassurance and validation from online communities, and feeling they had greater knowledge about their children's conditions. Some concerns and perceived risks were discussed, which often stemmed from prior unsatisfactory experiences of seeking information and support online, consultations with health professionals, and seeing distressing stories online.

Conclusion Most parents/carers were successful in obtaining information and support online. Many continued to share experiences with other parents/carers online. The need for information was particularly apparent early after diagnosis of the condition, whereas the need for peer-support continued. The potential concerns and perceived risks with information and support online were especially apparent amongst parents/carers of children with life-limiting long-term conditions. Findings may be useful for health professionals to facilitate discussions regarding use of online resources, and researchers designing online health resources for parents/carers.

PROSPERO registration number The protocol for this study was registered with PROSPERO in May 2018 [CRD42018096009].

For peer review only - http://bmjopen.bmj.com/site/about/guidelines.xhtml

Strengths and limitations of this study

- The first systematic review to synthesise the existing qualitative literature exploring parents/carers' experiences of seeking health information and support online for long-term physical childhood conditions
- A comprehensive study synthesising the experiences of hundreds of parents/carers of children with a broad range of long-term physical conditions which provides new insights and develops the research field
- A systematic and comprehensive search of the literature using multiple databases coupled with detailed synthesis of included studies
- Excludes the online experiences of parents/carers of children with acute, mental health, or developmental conditions, and otherwise healthy children
- Excluded quantitative studies which could have been used to support the qualitative findings

For peer review only

For peer review only - http://bmjopen.bmj.com/site/about/guidelines.xhtml

Background

Caring for a child with a long-term condition can be challenging for parents and carers, requiring ongoing management with regular treatment use, and often complicated, time-consuming and emotionally-laden everyday routines (1). Seeking to maintain treatment and care regimes, dealing with social and financial constraints, whilst balancing the needs of the whole family increase these challenges (2). For the remainder of the paper, parents and carers are collectively referred to as 'parents'.

As a source of health information, the Internet is convenient, instant and can help enhance knowledge and understanding of a condition (3). As a source of support, the Internet also enables parents to exchange stories with other parents with similar experiences. This commonly occurs through asynchronous discussion platforms (4), although synchronous (real-time) discussions through online platforms are also increasingly popular. Reviews of qualitative research have explored how the Internet influences peoples' health experiences (5, 6). Experiences include obtaining health information to understand illness, redressing offline information and knowledge deficits, discussing experiences with health services, building connections with others, feeling supported and validated, releasing emotions and offline frustrations, and learning self-management behaviours from others. Other reviews of qualitative research surrounding this area have explored the views of physicians regarding social media interventions to support child health (7), and experiences with health information online for carers of adults living with cancer (8). However, no systematic review has been conducted that synthesises the qualitative research on the online experiences of parents of children with long-term physical conditions.

Aim

This review aims to explore parents' experiences of seeking information and support online for long-term physical childhood conditions.

Method

This study follows The Centre for Reviews and Disseminations guidance for undertaking reviews in health care (9), and the ENTREQ guidelines for reporting the synthesis of qualitative research (10). The protocol for this study was registered with PROSPERO in May 2018 [CRD 2016018096009].

Eligibility criteria

We sought studies that explored parents' experiences of using the Internet for information and support about long-term physical childhood condition(s) (Table 1). To be eligible for inclusion, studies had to present qualitative data collection and analysis methods, and qualitative data. Studies that used a mixed methodology were included if they comprised a qualitative component substantive enough to perform a qualitative analysis.

	Inclusion criteria	Exclusion criteria

For peer review only - http://bmjopen.bmj.com/site/about/guidelines.xhtml

**Setting**	• Online health information and support resources (e.g. websites, forums, social media pages, blogs)	• Primarily offline health information and support resources (e.g. family, friends, books, leaflets)
**Design**	• Qualitative data collection methods (or mixed-methods studies involving qualitative data) (e.g. interviews), qualitative data analysis methods (e.g. thematic analysis) and presented qualitative data (e.g. themes, narratives and quotations)<br>• Primary research (e.g. full research papers, feasibility studies and pilot studies)	• Did not present the qualitative study criteria<br>• Not primary research (e.g. reviews, protocols and commentaries)
**Population**	• Parents and carers of children living with long-term physical health conditions	• Not parents or carers (e.g. family members without caring responsibility for the child, health professionals and teachers)
**Condition**	• Long-term physical health conditions<br>- Definition: A long-term or chronic physical health condition is a condition that requires ongoing management which cannot currently be cured but can be controlled with regular adherence to treatment (11)	• Acute conditions (e.g. common cold)<br>• Mental health conditions (e.g. depression)<br>• Developmental disorders of learning, language and cognition (e.g. Attention Deficit Hyperactivity Disorder)<br>• Healthy populations or health-promoting related studies (e.g. diet, physical activity, obesity)
**Outcome**	• Parents and carers' experiences	• Not parents or carers' experiences (e.g. patterns of Internet usage derived from quantitative studies)

Table 1. Eligibility criteria.

Search strategy

Five electronic databases were searched (Medline, CINAHL, Embase, PsycINFO, and The International Bibliography of the Social Sciences) from the earliest date possible to September 2019. The Cochrane Library of Systematic Reviews and PROSPERO were also searched to check for any ongoing similar reviews. A comprehensive search strategy was developed with a medical librarian from the University of Southampton for each database (Table 2). Search terms seeking to identify only qualitative research were initially included in search strategies, but we found that they did not improve the search results so were subsequently removed. Others have similarly found subject-specific search strategies rather than methodology-based strategies to be more effective in identifying all relevant studies reporting qualitative findings (12).

For peer review only - http://bmjopen.bmj.com/site/about/guidelines.xhtml

Database	Internet search terms	Parent/carer search terms	Use of online health information search terms	Paediatric search terms
**MEDLINE**	1. exp Internet/<br>2. forum*.mp.<br>3. facebook.mp.<br>4. social media.mp.<br>5. blog*.mp.<br>6. Internet.mp.<br>7. (web or online).mp.	1. parents/ or fathers/ or mothers/ or single parent/<br>2. Caregivers/<br>3. Legal Guardians/<br>4. (parent or parents).mp.<br>5. caregiver*.mp.<br>6. carer*.mp.<br>7. guardian*.mp.	1. Help-seeking Behaviour/<br>2. health information.mp.<br>3. Self-Help Groups/<br>4. self help.mp.<br>5. exp Health Education/<br>6. patient information.mp.<br>7. medical information.mp.<br>8. health information.mp.	1. Child Health/<br>2. Child/<br>3. Pediatrics/<br>4. (child or children or paediatric* or pediatric*).mp.
**CINAHL**	1. SU Internet<br>2. forum<br>3. facebook<br>4. social media<br>5. blog*<br>6. Internet	1. SU (parent* OR father* OR mother* OR single parent*)<br>2. SU caregivers<br>3. SU legal guardians<br>4. (parent OR parents)<br>5. caregiver*<br>6. carer*<br>7. guardian*	1. SU help seeking behaviour<br>2. health information<br>3. SU self help groups<br>4. self help<br>5. SU health education<br>6. patient information<br>7. medical information<br>8. health information	1. SU child health<br>2. SU child<br>3. SU paediatrics<br>4. (child OR children OR paediatric* OR pediatric*)
**Embase**	1. exp Internet/<br>2. forum*.mp.<br>3. facebook.mp.<br>4. social media.mp.<br>5. blog*.mp.<br>6. Internet.mp.<br>7. (web or online).mp	1. parents/ or fathers/ or mothers/ or single parent/<br>2. Caregivers/<br>3. Legal Guardians/<br>4. (parent or parents).mp.<br>5. caregiver*.mp.<br>6. carer*.mp.<br>7. guardian*.mp	1. Help-seeking Behaviour/<br>2. health information.mp.<br>3. Self-Help Groups/<br>4. self help.mp.<br>5. exp Health Education/<br>6. patient information.mp.<br>7. medical information.mp.<br>8. health information.mp.	1. Child Health/<br>2. Child/<br>3. Pediatrics/<br>4. (child or children or paediatric* or pediatric*).mp.
**PsycINFO**	1. SU Internet<br>2. forum<br>3. facebook<br>4. social media<br>5. blog*<br>6. Internet	1. SU (parent* OR father* OR mother* OR single parent*)<br>2. SU caregivers<br>3. SU legal guardians<br>4. (parent OR parents)<br>5. caregiver*<br>6. carer*<br>7. guardian*	1. SU help seeking behaviour<br>2. health information<br>3. SU self help groups<br>4. self help<br>5. SU health education<br>6. patient information<br>7. medical information<br>8. health information	1. SU child health<br>2. SU child<br>3. SU paediatrics<br>4. (child OR children OR paediatric* OR pediatric*)

For peer review only - http://bmjopen.bmj.com/site/about/guidelines.xhtml

For peer review only

**International Bibliography of Social Science**	1. Ab (Internet* OR forum* OR facebook OR social media OR blog* OR web* OR online)	1. Ab (parent* OR father* OR mother* OR caregiver* OR guardian* OR carer*)	1. Ab (help seeking behaviour* OR health information OR self help group* OR self help* OR health education OR patient information OR medical information)	1. Ab (child health OR child* OR paediatric* OR pediatric*)

Table 2. Search terms and syntax for each database.

For peer review only

For peer review only - http://bmjopen.bmj.com/site/about/guidelines.xhtml

Study screening methods

One author (BT) screened the titles and abstracts of all identified papers against the inclusion criteria. Duplicates of papers, and those that failed to meet the inclusion criteria were eliminated during this stage. A second author (ET) screened 20% of the titles and abstracts to check for consistency. Full papers for the remaining identified studies were retrieved and assessed for eligibility (see Figure 1).

Quality appraisal and data extraction

One author (BT) extracted information from the included studies and tabulated it. Assessors (BT, IM, MS, ET) independently assessed the quality of the included papers, using the Critical Appraisal Skills Programme (CASP) quality assessment tool for qualitative research (13). Any discrepancies were discussed with the research team.

Thematic Synthesis

We followed thematic synthesis for the analysis of the included studies (14), as this method has been successfully used for synthesising qualitative research in systematic reviews in health (15-20). Thematic synthesis consists of three stages. In stages 1 and 2 we explored and described the data. One author (BT) read and re-read included papers, then inductively coded each line of the studies' findings according to their content and meaning to develop an initial coding frame. Participants' original data excerpts, as well as the authors' descriptions of findings, were coded in the latest version of NVivo. The coding frame was then discussed and refined within the research team (BT, ET, IM, AR, NC, MS). In stage 3 we aimed to 'go beyond' individual study findings through developing descriptive themes initially and, subsequently, generating analytical themes.

Patient and Public Involvement

We involved an experienced public contributor with experience of eczema (AR) in the aim, design, analysis and write-up stages of the review. AR has a great deal of experience with online support groups, and has often highlighted that the online environment can be very supportive whilst also potentially bewildering. The contributor's experiences and preferences therefore helped shape the aim and design of the review: to explore parents' experiences of seeking information and support online for long-term physical childhood conditions.

Findings

Study selection

The database searches resulted in 2981 records after excluding duplicates. A final 23 (21-43) studies met all inclusion criteria and were included for thematic synthesis (Figure 1).

Study characteristics

All studies were published in English between December 2004 and July 2019, with the majority of studies (n = 14) published since April 2016. 11 studies were conducted in the Americas (USA (6), Canada (4), Brazil (1)), 10 studies were conducted in Europe (UK (5),

For peer review only - http://bmjopen.bmj.com/site/about/guidelines.xhtml

Sweden (2), The Netherlands (2), and Norway (1)), and 2 studies were conducted in Australia. The 23 studies presented the experiences of 559 parents captured through interviews and focus groups, 26 parents through free-text surveys and essays, and analyses of 2407 messages that had been posted by parents in online support groups (e.g. online forums, social media sites and blogs), though it should be noted that not all studies provided sample size information (Table 3).

For peer review only

For peer review only - http://bmjopen.bmj.com/site/about/guidelines.xhtml

Authors of study (Country) (ref.)	Aim(s)	Method of data collection (sample size)	Method of data analysis (as written by the authors)	Condition(s)
**Alsem et al., 2019 (The Netherlands) (21)**	To explore parental and physicians' experiences with the WWW-roadmap, as well as differences between parents who had used the WWW-roadmap to prepare for a consultation with their rehabilitation physician and parents who had not, in terms of empowerment and self-efficacy, parental and professional satisfaction with the consultation and family centredness of care.	Semi-structured interviews (sample size not stated)	Theoretical thematic analysis	Disabilities: Cerebral palsy , Neuromuscular, Metabolic disorder, Genetic disorder, Spina bifida, Various syndromes (e.g., Down's), Movement disorder, Unknown, Other * Included conditions that did not meet inclusion criteria.
**Benedicta et al., 2019 (Australia) (22)**	To understand how parents search for and appraise the relevance and credibility of online health information regarding their child, and how they use this information in order to help them make better health decisions for their child.	Think aloud task of searching online health info and semi-structured interviews (22)	Observation checklist for the think aloud task; inductive thematic analysis for the interviews	'Subacute' conditions * Potentially included conditions that did not meet inclusion criteria. Definition of 'subacute' not provided.
**Canty et al., 2019 (Canada) (23)**	To qualitatively and quantitatively assess the use of social media as an information-sharing and support-seeking tool by patients and caregivers**.**	Analysis of social media postings (sample size not stated)	Modified thematic analysis	Cerebral palsy
**Gage-Bouchard et al., 2019 (USA) (24)**	To examine the reasons paediatric cancer caregivers engage with cancer-related information on SNS and how caregivers appraise and assess the credibility of cancer-related information they encounter on this platform.	Semi-structured interviews (40)	Thematic analysis	Cancer
**Aston et al., 2018 (UK) (25)**	To explore the treatment-related experiences when children and young people take regular prescribed medication.	Semi-structured interviews (23 parents)	Thematic analysis	In-patients taking two or more prescribed medications concurrently at home, prior to admission, for 6 weeks or longer. Names of conditions not provided

For peer review only - http://bmjopen.bmj.com/site/about/guidelines.xhtml

				* Possibly included conditions that did not meet inclusion criteria.
**Hinton et al., 2018 (UK) (26)**	To explore parents' experiences and perspectives of having a baby who needs early abdominal surgery; identify the questions and problems that matter to parents during and after their pregnancy and infant's surgery and identify the long-term impact on parents and families.	Interviews (starting unstructured then semi-structured) (44)	Interpretative thematic analysis using a modified grounded theory approach, with constant comparison and exploration of deviant cases	Infants who needed abdominal surgery in the first year of life<br>* Included conditions that did not meet inclusion criteria.
**Holtz et al., 2018 (USA) (27)**	To develop a website resource, as an initial step in a larger intervention, for parents with a child with Type 1 Diabetes.	Focus groups (3 parents), interviews (3)	Iterative thematic analysis	Type 1 Diabetes
**Perez et al., 2018 (USA) (28)**	Stated as RQs: RQ1: What types of uncertainty do parents of a child with Type 1 Diabetes report experiencing?; RQ2: How do parents manage this uncertainty?	In-depth interviews (29)	Thematic analysis	Type 1 Diabetes
**Wright 2018 (USA) (29)**	To qualitatively explore the informal learning experiences of members of an online social media group hosted by Facebook.	Semi-structured interviews (25) and analyses of 604 postings (original and comments) created by members of the online social media	Several Grounded Theory methodologies	Gastro-oesophageal reflux disease
**Rehman et al., 2018 (Canada) (30)**	To establish the dominant themes of tweets posted by parents of children diagnosed with cancer and to situate the themes within a theoretical framework that describes the phenomenon of information sharing within a non-work context.	Analysis of the content of 1700 tweets from the Twitter accounts of 15 recruited participants	A deductive subjective interpretation	Cancer
**Tracey et al., 2018 (Australia) (31)**	To explore how parents acquire information to enhance their understanding of their child's disability and individualised funding schemes.	Focus groups (56 parents, 13 groups)	A a two stage, mixed method sequential approach which followed an initial survey	'disability'<br>* Possibly included conditions that did not meet inclusion criteria.
**Alsem et al., 2017 (The Netherlands) (32)**	To explore the experiences of Dutch parents of children with disabilities as regards their information needs, and to describe the process	Semi-structured interviews (15)	Open coding thematic analysis	Cerebral palsy, GMFCSa 2–5, rare disorders (e.g. genetic syndromes and metabolic and mitochondrial diseases)

For peer review only - http://bmjopen.bmj.com/site/about/guidelines.xhtml

	of seeking and evaluating information and the different sources of information.			* Possibly included conditions that did not meet inclusion criteria.
**Kirk & Milnes, 2016 (UK) (33)**	To explore how online peer support is used by young people and parents to support self-care in relation to cystic fibrosis.	Analysis of discussion group postings (103)	Inductive grounded theory	Cystic fibrosis
**Vogel et al., 2016 (Canada) (34)**	To explore parental usage of the National Kidney Foundation website, track visitor behaviour, evaluate usability and design, establish ways to improve user experience, and identify ways to redesign the website.	Mixed methods: Google analytic usage reports and focus groups (4)	Inductive content analysis and constant comparative approach	A condition requiring neurosurgery
**Santer et al., 2015 (UK) (35)**	To explore parents and carers' experiences of searching for information about childhood eczema on the Internet.	Semi-structured interviews (31)	Content analysis and constant comparative approach	Eczema
**Pimentel et al., 2013 (Brazil) (36)**	To describe the experience of patients with cystic fibrosis and their families using the Internet as a means for information, interaction and exchange of experiences with this disease.	Questionnaire (7)	Content analysis	Cystic fibrosis
**Nordfeldt et al., 2013 (Sweden) (37)**	To explore parents' views on their information-seeking, Internet use, and social networking online, and to identify implications for the future development of Internet use in this respect.	Focus groups (27)	Inductive qualitative content analysis	Type 1 Diabetes
**Gage & Panagakis, 2012 (USA) (38)**	To examine how parents think about, evaluate, access and use the Internet to seek information related to their child's cancer.	Mixed methods: Survey and structured interviews (41)	Grounded theory	Cancer
**Gundersen, 2011 (Norway) (39)**	To explore how the Internet can function as a resource that parents can draw on to cope with their situation.	Semi-structured interviews (10)	Not a specified method but looks like thematic analysis with comparative approach based on theorist	Rare genetic disorders that entail developmental and/or physical problems (e.g. allergies and epilepsy, and two had severe physical and learning problems)<br>* Included conditions that did not meet inclusion criteria.

For peer review only - http://bmjopen.bmj.com/site/about/guidelines.xhtml

**Stewart et al., 2011 (Canada) (40)**	To gather parents' preferences of support to design an innovative online peer support and test it.	Semi-structured interviews (44)	Deductive thematic content analysis	Asthma and allergies
**Nordfeldt et al., 2010 (Sweden) (41)**	To explore patients' and parents' attitudes toward a local Web 2.0 portal tailored to young patients with type 1 diabetes and their parents.	Essays (19)	Content analysis	Type 1 Diabetes
**Roche & Skinner, 2009 (USA) (42)**	To describe how parents of a child referred for genetic services searched the Internet for information, summarise how they interpreted and evaluated the information they obtained, and identify barriers that they encountered.	Semi-structured interviews (100)	Constant comparative method	Genetic
**Nettleton et al., 2004 (UK) (43)**	To explore parents and children's experiences of using the Internet for chronic childhood conditions.	Qualitative interviews (69)	Strategic comparisons approach'	Eczema, asthma, diabetes

Table 3. Summaries of the final 23 papers for thematic synthesis.

For peer review only - http://bmjopen.bmj.com/site/about/guidelines.xhtml

Quality of studies

The quality assessment of the included studies revealed that all studies met most quality indicators. All studies had clear statements of the aims of the research, qualitative methodology was appropriate in all studies and was collected in a way that addressed the aims. We did not exclude any studies based on quality of reporting.

Synthesis of results

The papers included in this review broadly focused on how parents of children with long-term physical conditions use online resources for health information and support, and what they gain (or not) from using online resources. By synthesising the papers' findings, we interpreted three main analytical themes: 1) Developing personal strategies for obtaining and appraising the online world; 2) Feeling supported and informed by the online world; 3) Perceived risk and concerns about the online world.

Figure 2 illustrates the analytical themes and descriptive themes, and table 4 lists all analytical and descriptive themes, along with references to the relevant papers.

For peer review only

For peer review only - http://bmjopen.bmj.com/site/about/guidelines.xhtml

Analytical theme	Descriptive theme	Paper references [total number]
**Developing personal strategies for obtaining and appraising the online world**		
	**Personal preferences**	22, 24, 27, 28, 29, 32, 33, 34, 35, 36, 37, 38, 39, 40, 41, 42, 43 **[17]**
	**Searching methods**	22, 24, 29, 31, 32, 34, 35, 37, 38, 39, 42, 43 **[12]**
	**Appraisal of trustworthiness and credibility**	22, 24, 27, 28, 29, 31, 32, 34, 35, 36, 37, 38, 43 **[13]**
	**Perceived need of information and support**	21, 22, 23, 26, 27, 28, 29, 33, 34, 35, 36, 37, 38, 39, 40, 41, 42, 43 **[18]**
	**Past positive experiences online**	21, 22, 24, 27, 28, 29, 31, 35, 36, 37, 38, 39, 40, 41, 42, 43 **[16]**
**Feeling supported and informed by the online world**		
	**Sense of community**	23, 24, 25, 26, 27, 28, 29, 30, 33, 35, 36, 37, 38, 39, 40, 43 **[16]**
	**Feeling informed**	21, 22, 23, 24, 25, 26, 27, 29, 30, 32, 33, 36, 37, 38, 39, 40, 41, 42, 43 **[19]**
**Concerns and perceived risks of the online world**		
	**Prior unsatisfactory experiences of seeking information and support online**	21, 22, 25, 28, 29, 31, 32, 34, 35, 36, 27, 38, 39, 41, 42, 43 **[16]**
	**Steered away by health professionals**	22, 26, 29, 32, 34, 38 **[6]**
	**Fear of what could find out online**	22, 25, 28, 29, 34, 38, 39, 42 **[8]**

Table 4. Overview of analytical and descriptive themes across the presenting papers.

For peer review only - http://bmjopen.bmj.com/site/about/guidelines.xhtml

How do parents develop personal strategies for obtaining and appraising information and support in the online world?

Parents were found to have **personal preferences** for certain types of websites and their content (22, 24, 27, 28, 29, 32, 33, 34, 35, 36, 37, 38, 39, 40, 41, 42, 43). Parents reported often favouring certain websites over others, which included official health information sites, charity websites, research sites, online discussion forums, and social media sites. From within the different types of websites, parents also expressed informational preferences including medically moderated content, personalised advice, and content dedicated to the condition without advertisements and other irrelevances. Parents also expressed functional preferences for online resources which included closed/private communities, live chat rooms, and notifications for new content. *"For me, this is a funnel. This group funnels you. People can get quick answers, but if you want more in-depth information, we always do the link to a website because it has way more in-depth information than what you can get in the group online."* (29; Gastro-oesophageal reflux disease, Interview and online forum study).

Parents also described a variety of **searching methods** which enabled them to obtain online information and support (22, 24, 29, 31, 32, 34, 35, 37, 38, 39, 42, 43)**.** Some visited the Internet with a specific question in mind, others reported typing in keywords that they had heard during their medical consultations, and others posted questions in a variety of online resources and combined the information with advice received offline. *"The same question on WhatsApp, Facebook and at the school"* (32; Various conditions including cystic fibrosis, metabolic conditions, other disabilities, Interview study).

Parents also reported **appraising the trustworthiness and credibility** of online resources, which influenced whether they decided to use the online resource (22, 24, 27, 28, 29, 31, 32, 34, 35, 36, 37, 38, 43). Some had reported more specific tactics such as matching up information from a variety of websites, checking the affiliation/source of the website or the background of individual posters, the possible sales and marketing intentions behind the websites, and comparing the advice with existing knowledge, whereas others implied that the reliability of online resources had been at least somewhat on their mind. *"[The website] that came up first, they've probably got advertisements in there… everything is paid, so I am a bit sus on that"* (22; Subacute conditions, Think aloud and interview study).

Parents' **perceived needs for information and support** also appeared to influence whether and how they searched for information and support online (21, 22, 23, 26, 27, 28, 29, 33, 34, 35, 36, 37, 38, 39, 40, 41, 42, 43). This involved the present stage and severity of their child's condition, such as a recent diagnosis or a change in symptoms, and an unsatisfying recent medical consultation. *"Five minutes after we got the diagnosis… we got on the computer. I didn't know what else to do."* (42; Genetic conditions, Interview study).

Finally, parents also expressed **past positive experiences with the online world**, and how these often encouraged continuous use (21, 22, 24, 27, 28, 29, 31, 35, 36, 37, 38, 39, 40, 41, 42, 43). Experiences included the Internet's convenient and accessible nature for obtaining

For peer review only - http://bmjopen.bmj.com/site/about/guidelines.xhtml

advice any time of the day, through a variety of mediums, for exploring sensitive topics and within the protection of anonymity, and general successes of obtaining desired information. *"You know when you post even though it's like 5:00 in the morning, you've got other people that are also awake and even though it is 5:00 in the morning, they respond as well within a timely manner."* (29; Gastro-oesophageal reflux disease, Interview and online forum study).

How and why do parents feel supported and informed by the online world?

The **sense of community** from using social media sites, discussion forums and blogs for peer-support was also emphasised (23, 24, 25, 26, 27, 28, 29, 30, 33, 35, 36, 37, 38, 39, 40, 43). Connecting with other parents with similar experiences online was described as comforting, and often reduced their loneliness and sorrow. The importance of building trust and relationships in online communities was also reported, which enabled parents to gain more advice from one another, to share common language, to be treated as equals, and to constantly be there to support one another. Parents also demonstrated that exchanging experiences online helped them to feel more reassured and validated about their situations. Also, parents expressed how online information and support that derives from others' experiences is invaluable, which often lacks in health professionals' advice. *"...I'm so happy that I found this page. For me to share & read about that there are other parents in my shoes. It breaks my heart watching my little girl try so hard to walk & play with other kids... Her condition has really taken a toll on me. Physically, mentally, & emotionally. I cry a lot. I'm just relieved there's parents on here that I can open up to."* (23; Cerebral palsy, Online forum study)

Parents across nearly all papers emphasised feeling **informed** through accessing and using information and support obtained online (21, 22, 23, 24, 25, 26, 27, 29, 30, 32, 33, 36, 37, 38, 39, 40, 41, 42, 43). The sense of feeling better informed was reported to derive from parents' development of condition-related knowledge including information about diagnosis, prognosis, triggers, and treatments. Parents often reported that their increase in medical knowledge helped them to feel in more control of managing their children's condition, and to cope with stress and during uncertain times such as changes in symptoms and treatments. Accessing information online was also reported to facilitate parents in subsequent medical consultations, through helping them to feel more equipped to understand what their health professionals were telling them, to structure questions, and to recommend plans for their children's treatments. Parents also reported feeling good about sharing their own experiences online about managing their children's conditions, such as with treatments, consultations, and coping methods. *"I think it allows you to then make a decision on how serious something might be or whether you need to take action straight away or if it's something that you can potentially wait up and see what happens"* (22; Subacute conditions, Think aloud and interview study).

How and why do parents develop concerns and perceive potential risks from seeking information and support online?

For peer review only - http://bmjopen.bmj.com/site/about/guidelines.xhtml

Whilst less common than positive accounts of feeling supported and informed, some parents reported the negative side to information and support online. Some parents reported that **prior unsatisfactory experiences of seeking information and support online** had discouraged them from using online resources (21, 22, 25, 28, 29, 31, 32, 34, 35, 36, 27, 38, 39, 41, 42, 43). Previous experiences included not being able to find their desired information and support, being overwhelmed by the volume of content online, feeling that the Internet does not replace real people, and a lack of digital literacy skills and experience. *"I choose not to [go online] because there are so many diverse opinions out there. There's so much garbage out there."* (38; Cancer, Survey and interview study).

Parents also reported experiences of being **steered away by health professionals** from using the Internet for information and support (22, 26, 29, 32, 34, 38). This was predominantly in papers that focused more on life-limiting conditions (e.g. needing surgery in first year of life, gastro esophageal reflux disease, cerebral palsy, neurological, cancer). *"[Physician B] was very adamant, "Don't you dare touch that Internet, do not look at it, do not— you listen to what I say, I'm the boss, and this is the way it's going to run… I just wanted the definition … I just wanted to know what the words meant…."* (34; Conditions requiring neurosurgery, Focus group study).

Finally, some parents reported that the **fear of what they could find out online** prevented them from using online resources for information and support (22, 25, 28, 29, 34, 38, 39, 42). Some reported having read distressing stories in the past, and others simply did not want to risk uncovering distressing information that they could relate to. These papers also focused more on life-limiting conditions (cancer, type 1 diabetes, rare genetic conditions, neurological). *"I was careful with going online because I think the internet can be a really scary place. We got into a couple of those forums that tend to be sort of doom and gloom and focus on all the negative things. It's all the people that are struggling the most that seem to post in some of those forums."* (28; Type 1 diabetes, Interview study).

Discussion

Summary of findings

This systematic review and thematic synthesis revealed that parents develop a variety of strategies to obtain and appraise information and support online in the context of caring for long-term physical childhood conditions. These processes were based on their personal preferences, their search methods, the outcome of their appraisal of trustworthiness, the degree of their perceived need for information and support, and previous positive experiences online. In most cases, parents reported going on to experience the benefits of accessing information and support online. This included the sense of community gained through connecting with others in a similar situation online, building relationships and trust, and feeling reassured and validated, which was available from online support communities especially. Parents also reported feeling better informed about their children's conditions, which helped them feel more in control in subsequent medical consultations and felt good

For peer review only - http://bmjopen.bmj.com/site/about/guidelines.xhtml

about helping similar others by sharing their own experiences. However, some parents also emphasised the concerns and perceived risks of obtaining and using information and support online. These often stemmed from prior unsuccessful online experiences, having been told to avoid the Internet by health professionals, and due to the fear of the distressing stories that they might come across online.

Findings in context of existing research

The findings from this study are largely consistent with other reviews that have explored peoples' experiences with health information online (5-6), although these reviews had not focused specifically on the online experiences of parents of children with long-term physical conditions. However our review uncovered more about the initial processes through which parents develop search strategies to obtain and evaluate health information and support online and the motivations behind these, which lead to the subsequent positive and negative experiences identified. Our review also drew more attention to the negative experiences that parents experience online compared to these reviews (5-6), which could be due to the additional challenges of caring for a child rather than self-caring.

Collating and synthesising the experiences of parents of children with a variety of long-term conditions provided additional insights into parents' experiences with the online world. Drawing on the different experiences discussed suggested that the potential harms of the online world were most apparent for parents of children with life-limiting conditions, or whose conditions were unstable (e.g. cerebral palsy, cancer, type 1 diabetes, rare genetic conditions, gastro-oesophageal reflux disease). Concerns and perceived risks were not found in the papers focusing on eczema, asthma and other allergies, and other disabilities. This suggests that parents of children with life-limiting or unstable conditions use information and support online with more caution.

This review was also able to further identify that the types of information and support sought by parents differed according to the stage and severity of their children's conditions. The need for condition-related information was again more apparent across the papers that focussed on the life-limiting or unstable conditions, whereas the need for peer-support was apparent across all stages and severities (life-limiting and uncertain to stable and well-managed). This suggests that parents' need for condition-specific information comes first and is not needed long-term, whereas the need for peer-support is continuous.

Strengths and Limitations

This is the first systematic review to synthesise the existing qualitative literature exploring parents' experiences of seeking health information and support online for long-term physical childhood conditions, providing new insights and developing the research field in this area.

A limitation to this study was that some of the included studies for synthesis also explored parents' online experiences for conditions that are not long-term and physical (e.g. acute conditions, disabilities of learning, language and cognition, mental health conditions, and health promotion in childhood), within the same paper. Some of the findings in these papers

For peer review only - http://bmjopen.bmj.com/site/about/guidelines.xhtml

were not separated according to different conditions, therefore this review fails to identify possible differences between the online experiences of parents of children with long-term physical conditions, and other types of conditions. However, this was never an aim of the study. Some included papers had limitations in reporting of methodological detail, for instance sample size information. Additionally, included studies typically presented limited demographic data so it was not possible to explore how such factors may affect parents' experiences in the online world. We also excluded quantitative studies which could have been used to support the qualitative findings, and did not search the grey literature which could have excluded some relevant studies. Finally, we analysed only the results sections of each paper, meaning that broader contextual factors that may have arisen in the discussion sections were not included although the discussion sections were read and checked in case.

Implications and future research

Improved understanding of parents' experiences of seeking health information and support online for long-term physical childhood conditions may help health professionals treating these families. The findings from this study could be used to inform health professionals about parents' experiences online: such as what they want from information and support online, why they choose online resources, what they are influenced by, and the types of information and support that they access online.

Parents' preferences and perceived benefits of online resources could be useful for future studies designing online resources for parents of children with long-term conditions. Specific examples that this study identified were online resources that contain medically moderated content, reminders to use online resources, frequently active members to reply to posts, easy to use, and information tailored to the child's condition and its severity. A future systematic review could collate which online resources for certain conditions are evidence-based and meet the needs of parents, so that health professionals can sign-post patients to such online resources.

Conclusion

This systematic review and synthesis of qualitative literature comprehensively explored parents' experiences with health information online for long-term physical childhood conditions. This highlights the support that parents receive from online resources, but also their perceptions of potential risks and the processes by which they attempt to establish trustworthiness of sources. These findings could help health professionals to understand parents' perspectives about information and support online, and potentially help them identify robust resources that also meet patients' needs.

For peer review only - http://bmjopen.bmj.com/site/about/guidelines.xhtml

For peer review only

References

1. Eiser C. Chronic childhood disease: An introduction to psychological theory and research. Cambridge University Press; 1990 Sep 27.27.

2. Smith J, Cheater F, Bekker H. Parents' experiences of living with a child with a long-term condition: a rapid structured review of the literature. Health Expectations. 2015 Aug;18(4):452-74.

3. Wainstein BK, Sterling-Levis K, Baker SA, Taitz J, Brydon M. Use of the Internet by parents of paediatric patients. Journal of paediatrics and child health. 2006 Sep;42(9):528-32.

4. Plantin L, Daneback K. Parenthood, information and support on the internet. A literature review of research on parents and professionals online. BMC family practice. 2009 Dec;10(1):1-2.

5. Ziebland SU, Wyke S. Health and illness in a connected world: how might sharing experiences on the internet affect people's health?. The Milbank Quarterly. 2012 Jun;90(2):219-49.

6. Allen C, Vassilev I, Kennedy A, Rogers A. Long-term condition self-management support in online communities: a meta-synthesis of qualitative papers. Journal of medical Internet research. 2016;18(3):e61.

7. Hamm MP, Shulhan J, Williams G, Milne A, Scott SD, Hartling L. A systematic review of the use and effectiveness of social media in child health. BMC pediatrics. 2014 Dec 1;14(1):138.

8. Kinnane NA, Milne DJ. The role of the Internet in supporting and informing carers of people with cancer: a literature review. Supportive Care in Cancer. 2010 Sep 1;18(9):1123-36.

9. Dissemination CF. Systematic reviews: CRD's guidance for undertaking reviews in healthcare. York: University of York NHS Centre for Reviews & Dissemination. 2009.

10. Tong A, Flemming K, McInnes E, Oliver S, Craig J. Enhancing transparency in reporting the synthesis of qualitative research: ENTREQ. BMC medical research methodology. 2012 Dec;12(1):181.

11. Department of Health and Social Care. Policy Paper: 2010 to 2015 government policy: long term health conditions. 2015. Accessed on 22 June 2020 from: https://www.gov.uk/government/publications/2010-to-2015-government-policy-long-term-health-conditions/2010-to-2015-government-policy-long-term-health-conditions#issue

12. Gorecki CA, Brown JM, Briggs M, Nixon J. Evaluation of five search strategies in retrieving qualitative patient-reported electronic data on the impact of pressure ulcers on quality of life. Journal of advanced nursing. 2010 Mar;66(3):645-52.

13. Critical Skills Appraisal Programme. CASP Qualitative Checklist. 2018.

14. Thomas J, Harden A. Methods for the thematic synthesis of qualitative research in systematic reviews. BMC medical research methodology. 2008 Dec 1;8(1):45.

For peer review only - http://bmjopen.bmj.com/site/about/guidelines.xhtml

15. Santer M, Ring N, Yardley L, Geraghty AW, Wyke S. Treatment non-adherence in pediatric long-term medical conditions: systematic review and synthesis of qualitative studies of caregivers' views. BMC pediatrics. 2014 Dec 1;14(1):63.

16. Morton RL, Tong A, Howard K, Snelling P, Webster AC. The views of patients and carers in treatment decision making for chronic kidney disease: systematic review and thematic synthesis of qualitative studies. BmJ. 2010 Jan 19;340:c112.

17. Joseph-Williams N, Elwyn G, Edwards A. Knowledge is not power for patients: a systematic review and thematic synthesis of patient-reported barriers and facilitators to shared decision making. Patient education and counseling. 2014 Mar 1;94(3):291-309.

18. Hefferon K, Grealy M, Mutrie N. Post-traumatic growth and life threatening physical illness: A systematic review of the qualitative literature. British journal of health psychology. 2009 May;14(2):343-78.

19. Pocock M, Trivedi D, Wills W, Bunn F, Magnusson J. Parental perceptions regarding healthy behaviours for preventing overweight and obesity in young children: a systematic review of qualitative studies. Obesity reviews. 2010 May;11(5):338-53.

20. Bridges J, Flatley M, Meyer J. Older people's and relatives' experiences in acute care settings: Systematic review and synthesis of qualitative studies. International journal of nursing studies. 2010 Jan 1;47(1):89-107.

21. Alsem MW, Verhoef M, Braakman J, van Meeteren KM, Siebes RC, Jongmans MJ, Visser-Meily JM, Ketelaar M, WWW, roadmap study group. Parental empowerment in paediatric rehabilitation: Exploring the role of a digital tool to help parents prepare for consultation with a physician. Child: Care, Health and Development. 2019 Sep;45(5):623-36.

22. Benedicta B, Caldwell PH, Scott KM. How parents use, search for and appraise online health information on their child's medical condition: A pilot study. Journal of Paediatrics and Child Health. 2020 Feb;56(2):252-8.

23. Canty MJ, Breitbart S, Siegel L, Fehlings D, Milo-Manson G, Alotaibi NM, Ibrahim GM. The role of social media in selective dorsal rhizotomy for children: information sharing and social support. Child's Nervous System. 2019 Nov 1;35(11):2179-85.

24. Gage-Bouchard EA, LaValley S, Devonish JA. Deciphering the Signal From the Noise: Caregivers' Information Appraisal and Credibility Assessment of Cancer-Related Information Exchanged on Social Networking Sites. Cancer Control. 2019 Apr 20;26(1):1073274819841609.

25. Aston J, Wilson KA, Terry DR. The treatment-related experiences of parents, children and young people with regular prescribed medication. International journal of clinical pharmacy. 2019 Feb 15;41(1):113-21.

26. Hinton L, Locock L, Long AM, Knight M. What can make things better for parents when babies need abdominal surgery in their first year of life? A qualitative interview study in the UK. BMJ open. 2018 Jun 1;8(6):e020921.

For peer review only - http://bmjopen.bmj.com/site/about/guidelines.xhtml

27. Holtz BE, Mitchell KM, Nuttall AK, Cotten SR, Hershey DD, Dunneback JK, Wood MA. Using user-feedback to develop a website: MyT1DHope, for parents of children with T1D. Health communication. 2020 Feb 23;35(3):281-8.

28. Perez L, Romo LK, Bell T. Communicatively Exploring Uncertainty Management of Parents of Children with Type 1 Diabetes. Health Communication. 2019 Jul 29;34(9):949-57.

29. Wright JV. Parents Caring, Sharing, and Learning Together Online: An Exploratory Look at Informal Learning via a Health-Related Support Group in Facebook. Arizona State University; 2018.

30. Rehman S, Lyons K, McEwen R, Sellen K. Motives for sharing illness experiences on Twitter: conversations of parents with children diagnosed with cancer. Information, Communication & Society. 2018 Apr 3;21(4):578-93.

31. Tracey D, Johnston C, Papps FA, Mahmic S. How do parents acquire information to support their child with a disability and navigate individualised funding schemes?. Journal of Research in Special Educational Needs. 2018 Jan;18(1):25-35.

32. Alsem MW, Ausems F, Verhoef M, Jongmans MJ, Meily-Visser JM, Ketelaar M. Information seeking by parents of children with physical disabilities: An exploratory qualitative study. Research in developmental disabilities. 2017 Jan 1;60:125-34.

33. Kirk S, Milnes L. An exploration of how young people and parents use online support in the context of living with cystic fibrosis. Health Expectations. 2016 Apr;19(2):309-21.

34. Vogel TK, Kleib M, Davidson SJ, Scott SD. Parental Evaluation of a Nurse Practitioner-Developed Pediatric Neurosurgery Website. JMIR research protocols. 2016;5(2):e55.

35. Santer M, Muller I, Yardley L, Burgess H, Ersser SJ, Lewis-Jones S, Little P. 'You don't know which bits to believe': qualitative study exploring carers' experiences of seeking information on the internet about childhood eczema. BMJ open. 2015 Apr 1;5(4):e006339.

36. Cargnin Pimentel ED, dos Santos Luz G, Marisa Pelloso S, Barros Carvalho MD. Using the internet to exchange information and experience on cystic fibrosis. Investigación y Educación en Enfermería. 2013 Dec;31(3):457-63.

37. Nordfeldt S, Ängarne-Lindberg T, Nordwall M, Krevers B. Parents of adolescents with type 1 diabetes-their views on information and communication needs and internet use. A qualitative study. PloS one. 2013 Apr 23;8(4):e62096.

38. Gage EA, Panagakis C. The devil you know: parents seeking information online for paediatric cancer. Sociology of health & illness. 2012 Mar;34(3):444-58.

39. Gundersen T. 'One wants to know what a chromosome is': the internet as a coping resource when adjusting to life parenting a child with a rare genetic disorder. Sociology of health & illness. 2011 Jan;33(1):81-95.

For peer review only - http://bmjopen.bmj.com/site/about/guidelines.xhtml

40. Stewart M, Letourneau N, Masuda JR, Anderson S, McGhan S. Online solutions to support needs and preferences of parents of children with asthma and allergies. Journal of Family Nursing. 2011 Aug;17(3):357-79.

41. Nordfeldt S, Hanberger L, Berterö C. Patient and parent views on a Web 2.0 Diabetes Portal—the management tool, the generator, and the gatekeeper: qualitative study. Journal of medical Internet research. 2010;12(2):e17.

42. Roche MI, Skinner D. How parents search, interpret, and evaluate genetic information obtained from the internet. Journal of genetic counseling. 2009 Apr 1;18(2):119-29.

43. Nettleton S, Burrows R, O'Malley L, Watt I. Health E-types' Information. Communication & Society. 2004;7(4):531-53.

For peer review only

For peer review only - http://bmjopen.bmj.com/site/about/guidelines.xhtml

Figures

Figure 1. PRISMA diagram of systematic search and study.

Figure 2. Illustration of the developed analytical themes and descriptive themes.

For peer review only

For peer review only - http://bmjopen.bmj.com/site/about/guidelines.xhtml

Contributors

Contributorship statement BT developed and refined the protocol, undertook the systematic review, data extraction, quality assessment, carried out the thematic synthesis, and drafted the manuscript. ET refined the protocol, contributed to the eligibility screening process, quality assessment, thematic synthesis, and writing the manuscript. MS and IM refined the protocol, contributed to quality assessment, thematic synthesis, and writing the manuscript. AR and NC refined the protocol, contributed to interpretation of findings, and writing the manuscript. All authors read and approved the final manuscript.

Funding This study was part a PhD studentship funded by the National Institute for Health Research (NIHR) School for Primary Care Research. The views expressed are those of the authors and not necessarily those of the NIHR or the Department of Health and Social Care.

Acknowledgements We would like to thank Paula Sands for her expertise in developing the search strategy for this study.

Competing interests None.

Data sharing No data are available.

For peer review only

For peer review only - http://bmjopen.bmj.com/site/about/guidelines.xhtml

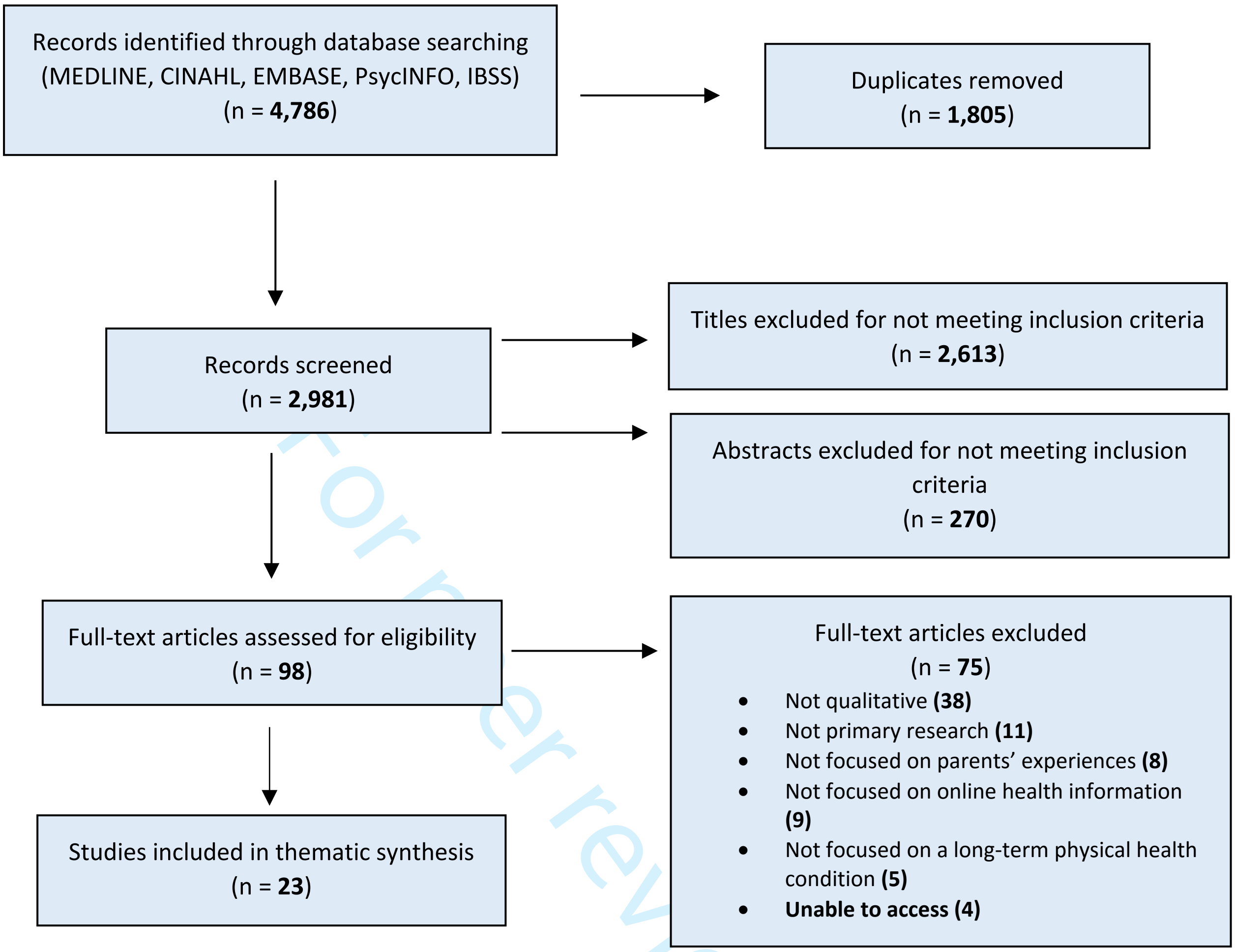

Figure 1. PRISMA diagram of systematic search and study.

For peer review only

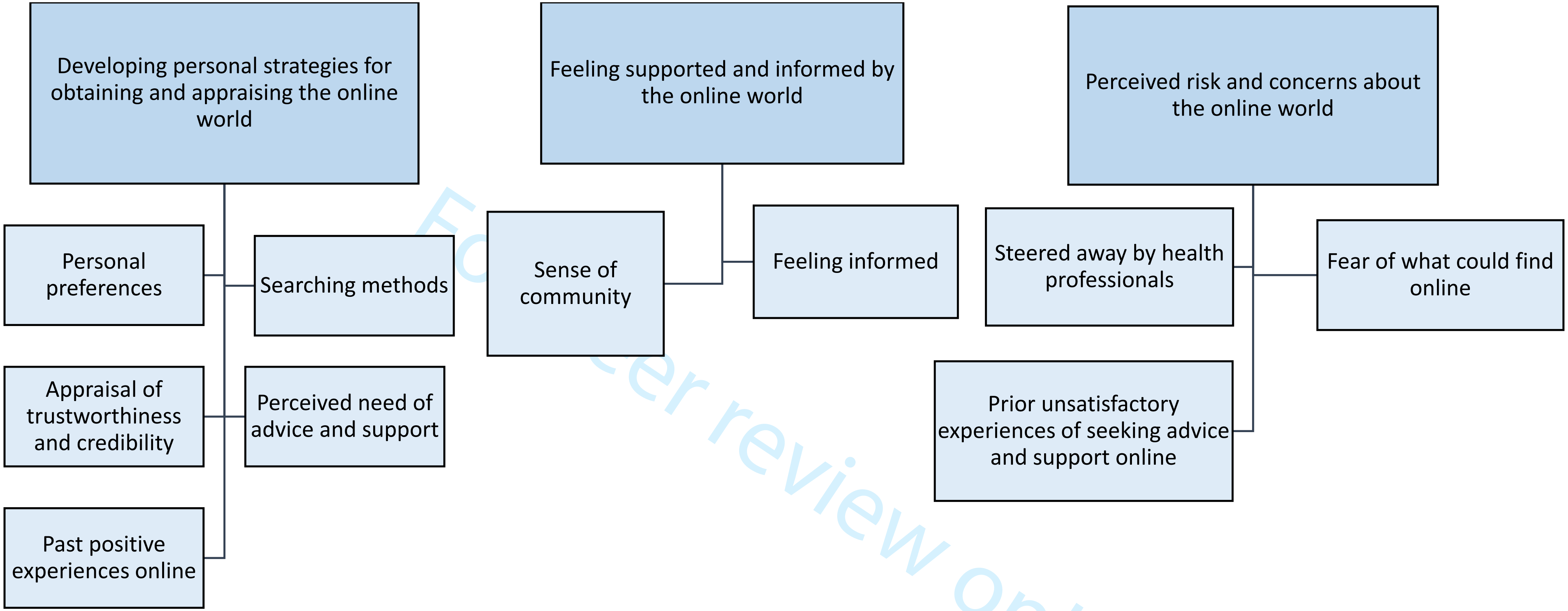

Figure 2. Illustration of the developed analytical themes and descriptive themes.

For peer review only

Reporting checklist for systematic review and meta-analysis.

Based on the PRISMA guidelines.

Instructions to authors

Complete this checklist by entering the page numbers from your manuscript where readers will find each of the items listed below.

Your article may not currently address all the items on the checklist. Please modify your text to include the missing information. If you are certain that an item does not apply, please write "n/a" and provide a short explanation.

Upload your completed checklist as an extra file when you submit to a journal.

In your methods section, say that you used the PRISMAreporting guidelines, and cite them as:

Moher D, Liberati A, Tetzlaff J, Altman DG, The PRISMA Group. Preferred Reporting Items for Systematic Reviews and Meta-Analyses: The PRISMA Statement

		Reporting Item	Page Number
**Title**			
	#1	Identify the report as a systematic review, meta-analysis, or both.	1
**Abstract**			
Structured summary	#2	Provide a structured summary including, as applicable: background; objectives; data sources; study eligibility criteria, participants, and interventions; study appraisal and synthesis methods; results; limitations; conclusions and implications of key findings; systematic review registration number	2
**Introduction**			
Rationale	#3	Describe the rationale for the review in the context of what is already known.	4

For peer review only

Objectives	#4	Provide an explicit statement of questions being addressed with reference to participants, interventions, comparisons, outcomes, and study design (PICOS).	4
**Methods**			
Protocol and registration	#5	Indicate if a review protocol exists, if and where it can be accessed (e.g., Web address) and, if available, provide registration information including the registration number.	4
Eligibility criteria	#6	Specify study characteristics (e.g., PICOS, length of follow-up) and report characteristics (e.g., years considered, language, publication status) used as criteria for eligibility, giving rational	4
Information sources	#7	Describe all information sources in the search (e.g., databases with dates of coverage, contact with study authors to identify additional studies) and date last searched.	5
Search	#8	Present full electronic search strategy for at least one database, including any limits used, such that it could be repeated.	6
Study selection	#9	State the process for selecting studies (i.e., for screening, for determining eligibility, for inclusion in the systematic review, and, if applicable, for inclusion in the meta-analysis).	8
Data collection process	#10	Describe the method of data extraction from reports (e.g., piloted forms, independently by two reviewers) and any processes for obtaining and confirming data from investigators.	8
Data items	#11	List and define all variables for which data were sought (e.g., PICOS, funding sources), and any assumptions and simplifications made.	n/a
Risk of bias in individual studies	#12	Describe methods used for assessing risk of bias in individual studies (including specification of whether this was done at the study or outcome level, or both), and how this information is to be used in any data synthesis.	n/a
Summary measures	#13	State the principal summary measures (e.g., risk ratio, difference in means).	n/a

For peer review only

Planned methods of analysis	#14	Describe the methods of handling data and combining results of studies, if done, including measures of consistency (e.g., I2) for each meta-analysis.	8
Risk of bias across studies	#15	Specify any assessment of risk of bias that may affect the cumulative evidence (e.g., publication bias, selective reporting within studies).	19
Additional analyses	#16	Describe methods of additional analyses (e.g., sensitivity or subgroup analyses, meta-regression), if done, indicating which were pre-specified.	n/a
**Results**			
Study selection	#17	Give numbers of studies screened, assessed for eligibility, and included in the review, with reasons for exclusions at each stage, ideally with a flow diagram.	8
Study characteristics	#18	For each study, present characteristics for which data were extracted (e.g., study size, PICOS, follow-up period) and provide the citation.	8
Risk of bias within studies	#19	Present data on risk of bias of each study and, if available, any outcome-level assessment (see Item 12).	n/a
Results of individual studies	#20	For all outcomes considered (benefits and harms), present, for each study: (a) simple summary data for each intervention group and (b) effect estimates and confidence intervals, ideally with a forest plot.	n/a
Synthesis of results	#21	Present the main results of the review. If meta-analyses are done, include for each, confidence intervals and measures of consistency.	14
Risk of bias across studies	#22	Present results of any assessment of risk of bias across studies (see Item 15).	n/a
Additional analysis	#23	Give results of additional analyses, if done (e.g., sensitivity or subgroup analyses, meta-regression [see Item 16]).	n/a
**Discussion**			
Summary of Evidence	#24	Summarize the main findings, including the strength of evidence for each main outcome; consider their relevance to	18

For peer review only

		key groups (e.g., health care providers, users, and policy makers	
Limitations	#25	Discuss limitations at study and outcome level (e.g., risk of bias), and at review level (e.g., incomplete retrieval of identified research, reporting bias).	19
Conclusions	#26	Provide a general interpretation of the results in the context of other evidence, and implications for future research.	20
**Funding**			
Funding	#27	Describe sources of funding or other support (e.g., supply of data) for the systematic review; role of funders for the systematic review.	26

The PRISMA checklist is distributed under the terms of the Creative Commons Attribution License CC-BY. This checklist was completed on 24. September 2020 using https://www.goodreports.org/, a tool made by the EQUATOR Network in collaboration with Penelope.ai

For peer review only

Bethan Treadgold supplementary file 1

Enhancing transparency in reporting the synthesis of qualitative research: the ENTREQ statement

From: Tong, A., Flemming, K., McInnes, E. *et al.* Enhancing transparency in reporting the synthesis of qualitative research: ENTREQ. *BMC Med Res Methodol* **12,** 181 (2012). https://doi.org/10.1186/1471-2288-12-181

No	Item	Guide and description	Page no.
**1**	Aim	State the research question the synthesis addresses.	4
**2**	Synthesis methodology	Identify the synthesis methodology or theoretical framework which underpins the synthesis, and describe the rationale for choice of methodology *(e.g. meta-ethnography, thematic synthesis, critical interpretive synthesis, grounded theory synthesis, realist synthesis, meta-aggregation, meta-study, framework synthesis).*	8
**3**	Approach to searching	Indicate whether the search was pre-planned (*comprehensive search strategies to seek all available studies)* or iterative (*to seek all available concepts until they theoretical saturation is achieved).*	5
**4**	Inclusion criteria	Specify the inclusion/exclusion criteria *(e.g. in terms of population, language, year limits, type of publication, study type).*	4
**5**	Data sources	Describe the information sources used (e.g. *electronic databases (MEDLINE, EMBASE, CINAHL, psycINFO, Econlit), grey literature databases (digital thesis, policy reports), relevant organisational websites, experts, information specialists, generic web searches (Google Scholar) hand searching, reference lists)* and when the searches conducted; provide the rationale for using the data sources.	5
**6**	Electronic Search strategy	Describe the literature search *(e.g. provide electronic search strategies with population terms, clinical or health topic terms, experiential or social phenomena related terms, filters for qualitative research, and search limits).*	6
**7**	Study screening methods	Describe the process of study screening and sifting *(e.g. title, abstract and full text review, number of independent reviewers who screened studies).*	8
**8**	Study characteristics	Present the characteristics of the included studies *(e.g. year of publication, country, population, number of participants, data collection, methodology, analysis, research questions).*	8

For peer review only

**9**	Study selection results	Identify the number of studies screened and provide reasons for study exclusion *(e,g, for comprehensive searching, provide numbers of studies screened and reasons for exclusion indicated in a figure/flowchart; for iterative searching describe reasons for study exclusion and inclusion based on modifications t the research question and/or contribution to theory development).*	8
**10**	Rationale for appraisal	Describe the rationale and approach used to appraise the included studies or selected findings *(e.g. assessment of conduct (validity and robustness), assessment of reporting (transparency), assessment of content and utility of the findings).*	8
**11**	Appraisal items	State the tools, frameworks and criteria used to appraise the studies or selected findings *(e.g. Existing tools: CASP, QARI, COREQ, Mays and Pope* [25]*; reviewer developed tools; describe the domains assessed: research team, study design, data analysis and interpretations, reporting).*	8
**12**	Appraisal process	Indicate whether the appraisal was conducted independently by more than one reviewer and if consensus was required.	8
**13**	Appraisal results	Present results of the quality assessment and indicate which articles, if any, were weighted/excluded based on the assessment and give the rationale.	14
**14**	Data extraction	Indicate which sections of the primary studies were analysed and how were the data extracted from the primary studies? *(e.g. all text under the headings "results /conclusions" were extracted electronically and entered into a computer software).*	8
**15**	Software	State the computer software used, if any.	8
**16**	Number of reviewers	Identify who was involved in coding and analysis.	8
**17**	Coding	Describe the process for coding of data *(e.g. line by line coding to search for concepts).*	8
**18**	Study comparison	Describe how were comparisons made within and across studies *(e.g. subsequent studies were coded into pre-existing concepts, and new concepts were created when deemed necessary).*	8
**19**	Derivation of themes	Explain whether the process of deriving the themes or constructs was inductive or deductive.	8
**20**	Quotations	Provide quotations from the primary studies to illustrate themes/constructs, and identify whether the quotations were participant quotations of the author's interpretation.	16
**21**	Synthesis output	Present rich, compelling and useful results that go beyond a summary of the primary studies (e.g. *new interpretation, models of evidence, conceptual models, analytical framework, development of a new theory or construct).*	16

For peer review only
